# Pre-treatment soluble PD-L1 as a predictor of overall survival for immune checkpoint inhibitor therapy: a systematic review and meta-analysis

**DOI:** 10.1007/s00262-022-03328-9

**Published:** 2022-11-16

**Authors:** Ádám Széles, Tamás Fazekas, Szilard Váncsa, Melinda Váradi, Petra Terézia Kovács, Ulrich Krafft, Viktor Grünwald, Boris Hadaschik, Anita Csizmarik, Péter Hegyi, Alex Váradi, Péter Nyirády, Tibor Szarvas

**Affiliations:** 1 Department of Urology, Semmelweis University, Üllői Út 78/B, Budapest, 1082 Hungary; 2 Center for Translational Medicine, Semmelweis University, Budapest, Hungary; 3 Institute for Translational Medicine, Szentágothai Research Centre, Medical School, University of Pécs, Pécs, Hungary; 4 Division of Pancreatic Diseases, Heart and Vascular Center, Semmelweis University, Budapest, Hungary; 5 Department of Urology, University of Duisburg-Essen and German Cancer Consortium (DKTK)-University Hospital Essen, Hufelandstraße 55, 45147 Essen, Germany

## Abstract

**Introduction:**

Immune checkpoint inhibitors (ICI) such as anti-PD-L1 and anti-PD-1 agents have been proven to be effective in various cancers. However, the rate of non-responders is still high in all cancer entities. Therefore, the identification of biomarkers that could help to optimize therapeutic decision-making is of great clinical importance. Soluble PD-L1 (sPD-L1) and PD-1 (sPD-1) are emerging blood-based biomarkers and were previously shown to be prognostic in various clinical studies.

**Objective:**

We aimed to evaluate the prognostic relevance of sPD-L1 and sPD-1 in patients with different tumor entities who underwent ICI therapy.

**Methods:**

We searched for articles in PubMed via Medline, Embase, Scopus, and Cochrane databases. The primary outcome was overall survival (OS) and progression-free survival (PFS); furthermore, we analyzed on-treatment serum level changes of sPD-L1 and sPD-1 during ICI therapy.

**Results:**

We synthesized the data of 1,054 patients with different cancer types from 15 articles. Pooled univariate analysis showed that elevated levels of sPD-L1 were significantly associated with inferior OS (HR = 1.67; CI:1.26–2.23, *I*^2^ = 79%, *p* < 0.001). The strongest association was found in non-small cell lung cancer, whereas weaker or no association was observed in melanoma as well as in renal cell and esophageal cancers. Pooled multivariate analysis also showed that elevated levels of sPD-L1 correlated with worse OS (HR = 1.62; CI: 1.00–2.62, *I*^2^ = 84%, *p* = 0.05) and PFS (HR = 1.71; CI:1.00–2.94, *I*^2^ = 82%, *p* = 0.051). Furthermore, we observed that one or three months of anti-PD-L1 treatment caused a strong (27.67-fold) elevation of sPD-L1 levels in malignant mesothelioma and urothelial cancer.

**Conclusions:**

We found significantly inferior OS in ICI-treated cancer patients with elevated pre-treatment sPD-L1 levels, but this association seems to be tumor type dependent. In addition, sPD-L1 increases during anti-PD-L1 therapy seems to be therapy specific.

**Supplementary Information:**

The online version contains supplementary material available at 10.1007/s00262-022-03328-9.

## Introduction

Recently, growing evidence suggests that immune checkpoint inhibition with both programmed death protein-1 (PD-1) and programmed death protein ligand-1 (PD-L1) inhibitors is effective therapeutic options for several cancer types. Immune checkpoint inhibitors (ICI) revolutionized anti-cancer therapy, but the rate of non-responders is still high and varies significantly between various cancers [1–4]. Therefore, there is a great clinical need for prognostic and predictive biomarkers to identify patients who will respond to ICI therapy. Currently, ICIs are widely used in non-small cell lung cancer (NSCLC), melanoma, renal cell carcinoma (RCC), urothelial cancer, and breast cancer, but the list of indications is rapidly expanding [5].

PD-1 and PD-L1 are membrane-bound co-inhibitory immune checkpoint receptors expressed by various human immune and cancer cells. PD-1 is primarily located in T-cells, whereas PD-L1 is most abundantly expressed by cancer cells. Binding between PD-L1 and its receptor PD-1 leads to immune suppression, which helps cancer cells to escape from cytotoxic T-cell-mediated lysis [6]. PD-1 and PD-L1 are not only found on the surface of cells, but their soluble forms can be detected in blood circulation both in healthy individuals and cancer patients. Similar to its membrane-bond tissue expression, elevated serum PD-L1 (sPD-L1) and PD-1 (sPD-1) levels were generally associated with more advanced disease stages and worse survival, suggesting that these serum markers are prognostic in various tumors [7–11]. However, their predictive value regarding various systemic treatments remained largely contradictory.

There are only few available biomarkers for ICI therapy, such as tissue PD-L1 immunohistochemistry (IHC), tumor mutational burden, or microsatellite instability. However, their predictive ability is different among various tumor types.[12, 13]. For example, PD-L1 immunohistochemistry shows predictive value in NSCLC, head and neck squamous cell (HNSCC), and urothelial cancer [14–16], whereas in melanoma and RCC, PD-L1 IHC cannot be used for the prediction of ICI therapy [17, 18]. In addition, tissue-based IHC has further limitations related to the heterogeneity of PD-L1 expression, different characteristics of various diagnostic antibodies, and differences in evaluation methods [19]. Furthermore, as repeated biopsy for follow-up purposes is hardly feasible, tissue analysis is much less suitable for therapy monitoring than serum-based assays. Therefore, an unmet clinical need is the application of easily accessible, blood-based biomarkers determined by an easy-to-use and robust analytical method for pre-treatment prediction and monitoring of ICI therapy.

In the present study, we conducted a systematic review and meta-analysis of published literature data to assess the prognostic significance of circulating sPD-L1 and sPD-1 levels in pre-treatment and on-treatment samples of tumor patients who underwent ICI therapy.

## Methods

The study was reported according to the Preferred Reporting Items for Systematic Reviews and Meta-analyses (PRISMA) 2020 recommendations [20], and the Cochrane Handbook was followed [21]. The protocol was registered on PROSPERO (Nr.:CRD42021283222).

### Literature search

Electronic databases from PubMed, Scopus, Embase, and Cochrane library were screened to identify studies investigating the prognostic role of sPD-L1 and sPD-1 in various cancers treated with ICIs. Additionally, references of included studies were screened to identify further potentially eligible studies. Two independent authors (AS and TF) performed the systematic search and the selection process. References were screened using EndNote X^9^ (Clarivate Analytics, Philadelphia, PA, USA) and assessed by title, abstract, and full text.

### Eligibility criteria

The PECO framework was applied to state our research question. We included original studies in the English language, which investigated (P) ICI-treated patients with various tumors, and (E and C) compared the hazard of high and low serum or plasma sPD-L1 and/or sPD-1 levels in regard to (O) overall survival (OS) or progression-free survival (PFS). There was no pre-defined cut-off value for the definition of high and low levels of biomarkers. If available, on-treatment sPD-L1 and sPD-1 concentrations (median or mean level, range, or interquartile range) were also considered as additionally assessed parameters.

The following exclusion criteria were used: study design: reviews, comments, letters, meta-analysis, systematic reviews, animal experiments, and conference abstracts. No restrictions were made regarding cohort size and study design.

### Data extraction

Two independent authors (AS, TF) extracted data by reading full-text articles. Extracted parameters were the following: the first name of the author, year of publication, cancer type, ICI therapy type, country of sample/data collection, study type, cohort size, patient age, sex, cut-off values for sPD1/sPD-L1, cut-off definition method (e.g., median, receiver operating characteristic curve—ROC), assay method, follow-up time, OS, and PFS.

In eligible studies, either the article provided calculated hazard ratios (HR) with a 95% Confidence Interval (CI), or the overall HR and 95% CI were estimated from Kaplan–Meier curves by using the GetData Graph Digitizer software v2.26™. In addition, when available, data on changes of sPD-L1 and sPD-1 during ICI treatment were extracted.

### Quality assessment and evaluation of evidence

**Fig. 1:**
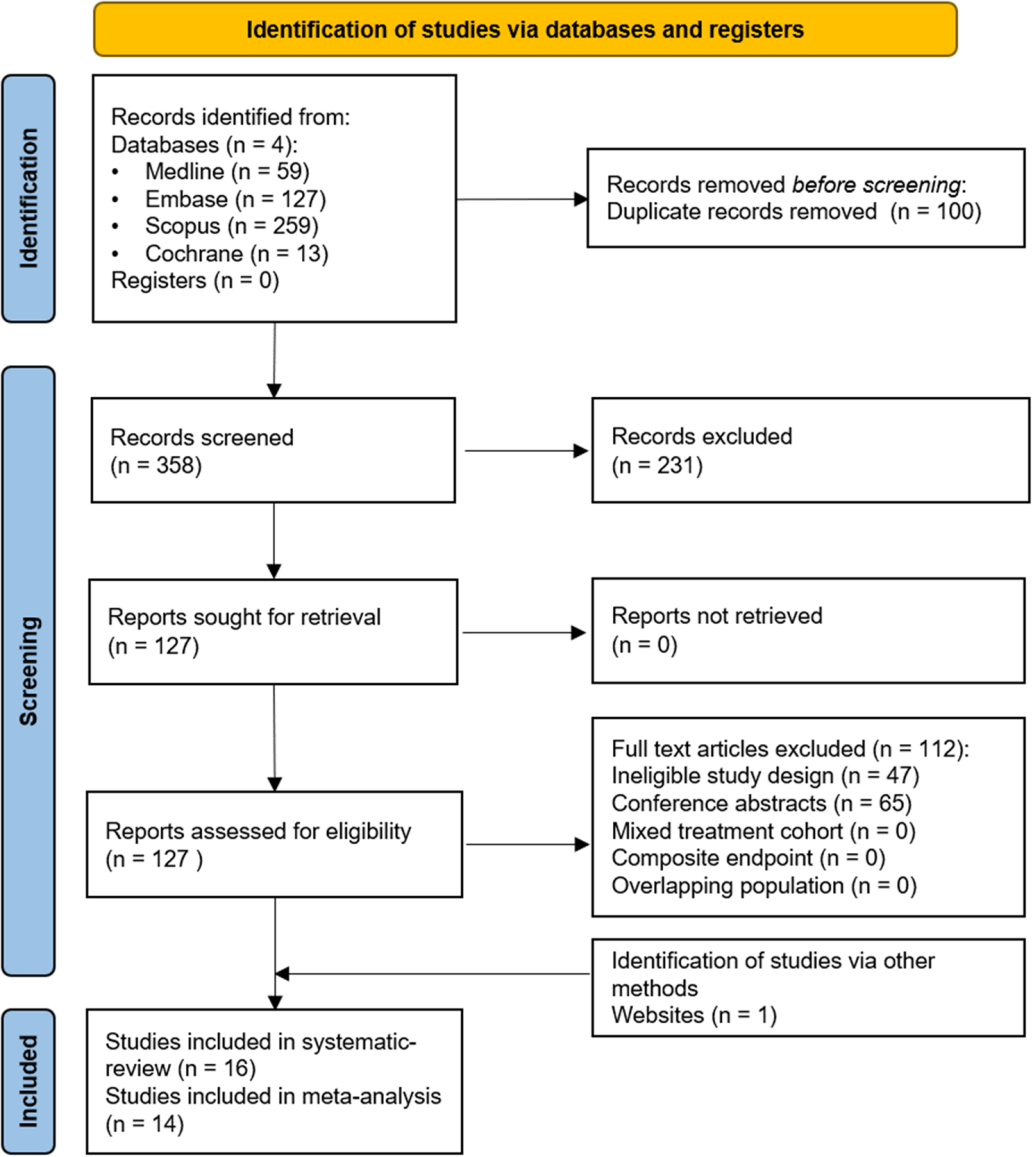
PRISMA 2020 flowchart representing the study selection process

Two independent authors performed the risk of bias assessment using the Quality in Prognostic Studies (QUIPS) tool [22]. The study attrition domain was assessed only in the case of prospective studies. We used the RobVisR tool to summarize the results of the assessments [23]. (Supplementary Table 1, Fig. 1) For the level of evidence assessment, the GRADEpro™ program [24]. (Supplementary Table 2) was applied.

### Synthesis methods

Random-effects models with the inverse variance method were applied to pool hazard ratios (HR) with 95% confidence interval (CI) in the case of all outcomes. For the outcomes where the study number was over 5, a Hartung–Knapp adjustment [25, 26] was applied. The restricted maximum-likelihood method [27] was used to estimate variance measure 2τ2, and between-study heterogeneity was investigated with Cochrane Q test and the Higgins & Thompson’s I2I2 statistics [28]. The Q test was considered significant when the p value was less than 0.1. Forest plots were used to graphically summarize the results. Where applicable we reported the prediction intervals of results according to IntHout et al. [29]. Outlier and influence analyses were carried out following the recommendations of Harrer [27] and Viechtbauer and Cheung [30]. Small study effect was investigated on funnel plots, and if there had been at least 10 studies, it would have been assessed statistically using Egger’s test. Subgroup analysis was conducted based on the used ELISA assays and cancer types. In the case of subgroup analysis, a fixed-effects “plural” model was applied (aka. mixed-effects model). To assess the difference between the subgroups, Cochrane Q test was used [27]. The null hypothesis was rejected at a 5% significance level. All statistical analyses were performed with R [31] statistical environment and language, using the *meta* [32] and *dmetar* [33] packages. P < 0.05 was considered significant. Biomarker level changes were expressed as fold-changes, and a median fold-change was calculated separately for PD-1 and PD-L1 inhibitors (Table 2).

## Results

### Search and selection

Using the above-defined search key, 458 articles were initially retrieved from the accessed databases (Fig. 1). After the selection process, 16 articles matched our eligibility criteria [10, 34–48]. However, the HR and 95% CI estimation in two articles were not possible [34, 35]. Therefore, these two articles were included only in the qualitative synthesis.

### Baseline characteristics of included studies

Baseline characteristics of the included articles are summarized in Table 1. Cancer types included in this systematic review were the following: NSCLC, RCC, melanoma, esophageal squamous cell cancer (ESCC), urothelial cancer, and mesothelioma. Nine articles reported the results of prospectively performed studies, and seven articles were retrospective. Only three articles included data on sPD-1, and all the included publications reported on sPD-L1. Different studies used different strategies to set cut-off values; six studies used the median as cut-off, while eight used the ROC analysis to adjust the cut-off values. All but one study used the ELISA assay technique to determine sPD-1 or sPD-L1 concentrations. Nine studies applied ELISA assays by R&D Systems (Wiesbaden, Germany), and other studies used assays by Cloud-Clone (Dynabio, Marseille, France), Abcam (Cambridge, UK), Invitrogen (Thermo Fisher, Darmstadt, Germany), and SIMOA (Billerica, MA, USA). The remaining article used a multiplex immunoassay (14-ProcartaPlex Human Immuno-Oncology Checkpoint Panel by Invitrogen (Thermo Fisher, Darmstadt, Germany)). Ten articles reported on-treatment sPD-1/sPD-L1 levels in addition to baseline levels [10, 34–38, 40, 42, 45, 47].

**Table 1:** Basic characteristics of included studies

Author (year)	Type of cancer treatment	Study site/type	No. of patients (female %)	Age (year)	Biomarker/Cut-off(pg/mL)/type of cut-off	Follow-up period (months)	Type of ELISA	Outcome
Ando et al. 2019^†^ [34]	NSCLC—Nivo/ Pembro Gastric—Nivo/ Pembro Bladder—Pembro	Japan/R	21 (29)	NA	sPD-L1/347/median	6.0 (1.0–27.0)	R&D	OS
Castello et al. 2020^†^ [35]	NSCLC—mixed	Italy/P	20 (35)	77 (51–86)	sPD-L1/27.2/median	10.3 (2.0–29.0)	R&D	OS/PFS
Chiarucci et al. 2020 [36]	Mesothelioma—Durva/Termeli	Italy/P	40 (34)	66 (42–83)	sPD-L1/70/median	19.2 (13.8 – 20.5) ^*^	R&D	OS
Costantini et al. 2018 [37]	NSCLC—Nivo	France/R	43 (33)	68 (62–72)	sPD-L1/34/ROC	16.3 (11.7–21.1) ^*^	Abcam	OS/PFS
Incorvaia et al*.* 2020 [38]	RCC—Nivo	Italy/P	21 (10)	61 (36–70)	sPD-L1/660/ROC sPD-1/2110/ROC	NA	DYNABIO	PFS
Ji et al. 2020 [39]	ESCC—anti-PD-1 Mixed–mixed	China/R	21 (5) 61 (39)	57 (46–70) 43 (21–64)	sPD-L1/NA/ROC	NA	Multiplex immunoassay kit	OS/PFS
Krafft et al. 2021^#^ [10]	Urothelial—Atezo/Pembro	Hungary/P	19 (26)	66 (43 – 77)	sPD-L1/76/median	17.0 (6.0–31.0)	R&D	OS/PFS
Mahoney et al. 2021 [40]	RCC—Nivo Melanoma—Nivo	USA/P	91 (33) 78 (44)	NA	sPD-L1/NA/NA	NA	SIMOA	OS/PFS
Mazzaschi et al*.* 2020 [41]	NSCLC—Nivo/Pembro/Atezo	Italy/P	109 (33)	72 (41–85)	sPD-L1/113/CART tree	17.3	R&D	OS/PFS
Meyo et al. 2020 [42]	NSCLC—Nivo	France/P	51 (43)	66 (60 – 69)	sPD-L1/160/median sPD-1/70/median	26.4 (18.1 – 36.5)	Cloud-Clone	OS/PFS
Murakami et al*.* 2020 [43]	NSCLC—Pembro/Nivo	Japan/R	233 (35)	63 (30–84)	sPD-L1/90/mean + 2sd	NA	R&D	OS/PFS
Okuma et al. 2018 [44]	NSCLC—Nivo	Japan/P	39 (26)	69 (50–88)	sPD-L1/3357/ROC	NA	Cloud-Clone	OS
Oh et al. 2021 [45]	Mixed–mixed	Korea/R	128 (31)	62 (21–82)	sPD-L1/11/ROC	NA	Invitrogen	OS/PFS
Ugurel et al*.* 2019 [46]	Melanoma—anti-PD-1	Germany/R	85 (41)	62^**^	sPD-L1/10/ROC sPD-1/500/ROC	12.1	R&D	OS/PFS
Yang et al. 2021 [47]	NSCLC—mixed	China/P	21 (NA)	NA	sPD-L1/0.95^##^/fold change	NA	R&D	OS/PFS
Zhou et al. 2017 [48]	Melanoma—Pembro	USA/NA	35 (NA)	NA	sPD-L1/1400/NA	NA	R&D	OS

Nivo—nivolumab, Pembro—pembrolizumab, Termeli—tremelimumab, Durva—durvalumab, Atezo—atezolizumab, NSCLC—non-small cell lung cancer, RCC—renal cell carcinoma, ESCC—esophageal squamous cell carcinoma, R—retrospective, P—prospective, ROC—receiver operating curve, CART—Classification and Regression Trees, sd—standard deviation, OS—overall survival, PFS—progression-free survival *IQR, **mean, ^†^ included only in systematic review, ^#^ cohort data are supplemented with unpublished data, ^##^ Fold change

### Elevated pre-treatment sPD-L1 predicts OS in NSCLC and melanoma

**Fig. 2:**
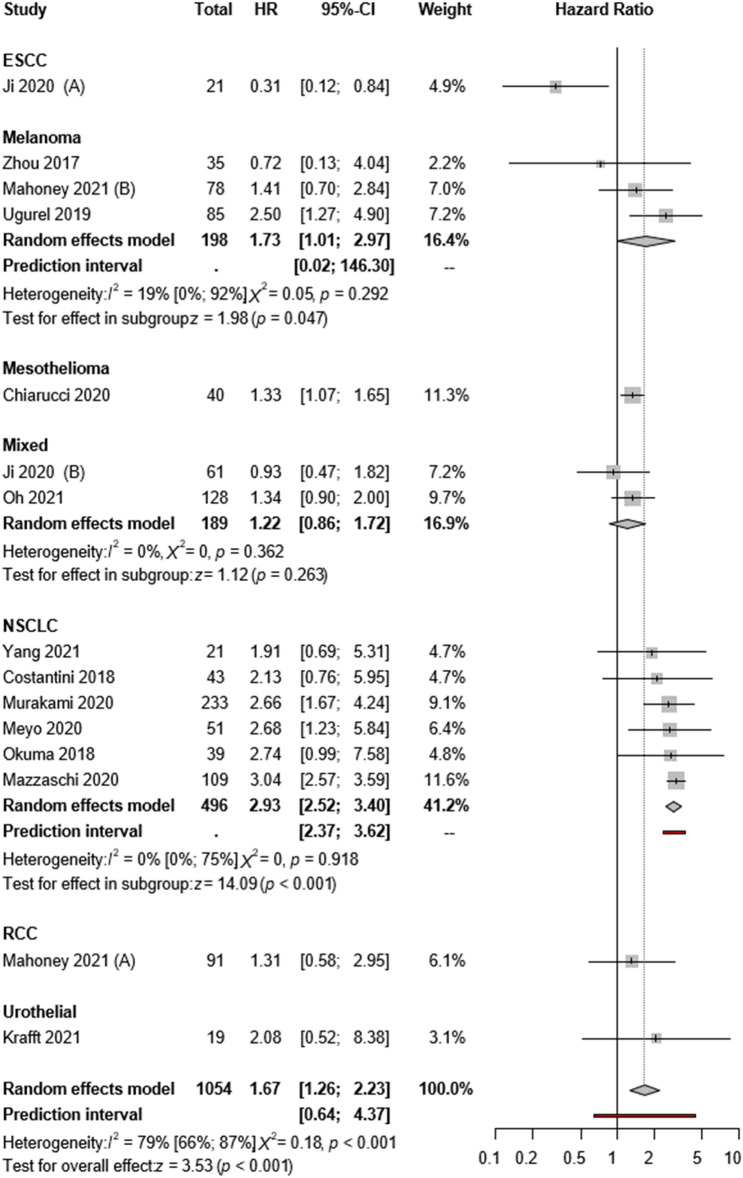
Forest plots representing hazard ratios of OS for sPD-L1 in different tumor types

Thirteen articles reported univariate OS as a primary outcome. The pooled overall estimate showed that patients with high sPD-L1 levels had worse OS (HR:1.67; CI:1.26–2.23, *I*^2^ = 79%, *p* < 0.001; Fig. 2). As for publication bias, the funnel plot seems asymmetric; however, Egger’s test shows no publication bias (*p* = 0.177)(Supplementary Figs. 5 and 7).

Four of the included articles reported a multivariate Cox proportional hazard model. The pooled multivariate analysis confirmed that patients with high sPD-L1 levels had shorter OS (HR:1.62; CI:1.00–2.62, *I*^2^ = 84%, *p* = 0.05; Supplementary Fig. 2).

A subgroup analysis was performed according to cancer type. Based on six studies with NSCLC patients, high sPD-L1 levels were consequently associated with poor OS (HR:2.93; CI:2.52–3.40, *I*^2^ = 0%, *p* < 0.001). According to three publications, poorer OS was found for malignant melanoma (HR:1.73; CI:1.01–2.97, *I*^2^ = 19% *p* = 0.047). No difference was found between high and low sPD-L1 levels in OS in the subgroup of mixed tumor types (HR:1.22; CI:0.86–1.72, *I*^2^ = 0%, *p* = 0.263), but in this case, various studies showed rather heterogeneous results (Fig. 2).

### Elevated pre-treatment sPD-L1 predicts poor PFS in NSCLC

**Fig. 3:**
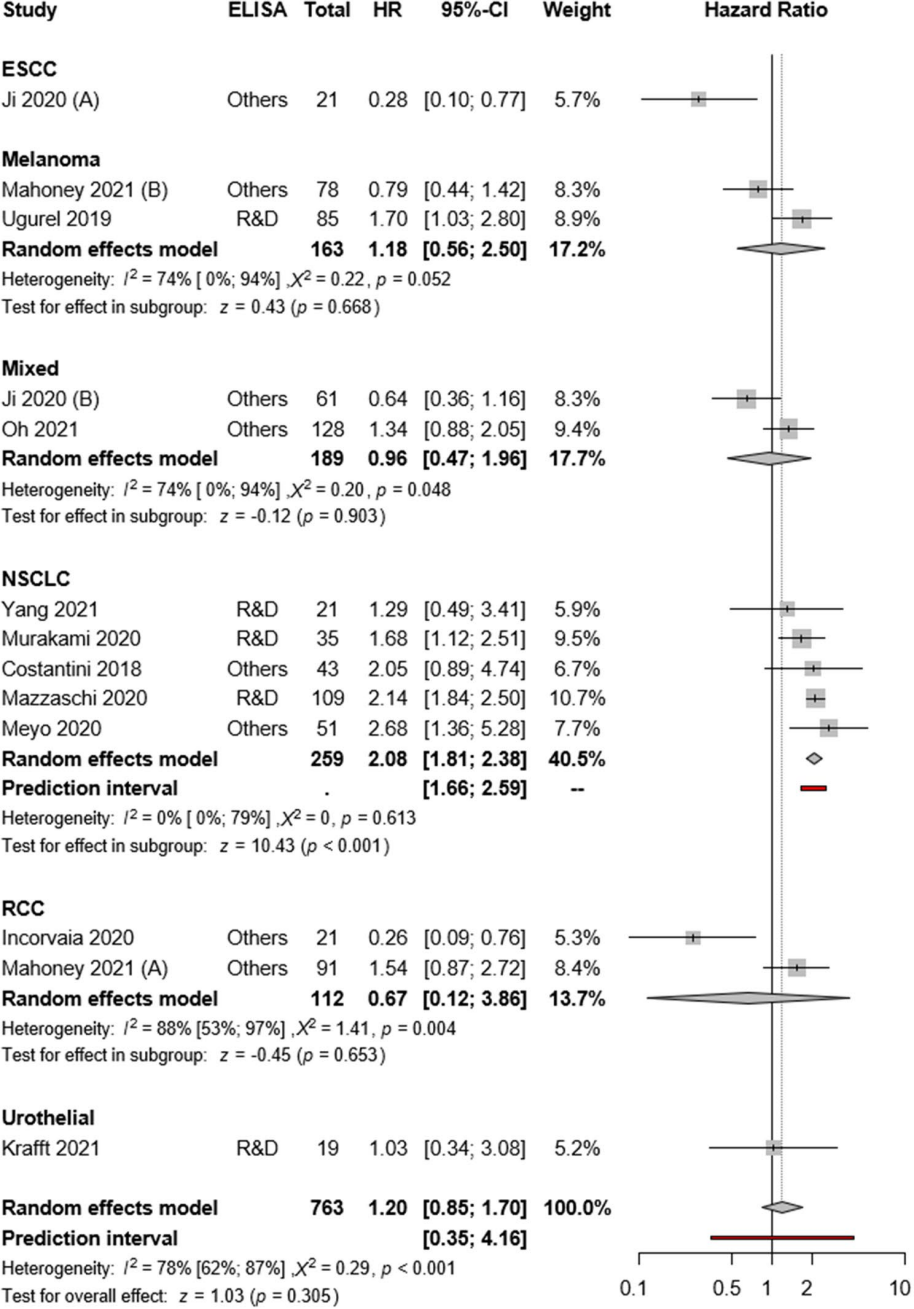
Forest plots representing hazard ratios of PFS for sPD-L1 in different tumor types

Eleven articles reported univariate PFS as the primary outcome. The pooled overall estimate found no PFS difference between high and low sPD-L1 groups (HR:1.20; CI:0.85–1.70, *I*^2^ = 78%, *p* = 0.305; Fig. 3). The visual presentation of the Funnel plot and Egger’s test suggested publication bias (*p* = 0.007) (Supplementary Figs. 6 and 8).

Four of the included articles reported a multivariate Cox proportional hazard model. The pooled multivariate analysis showed that patients with high sPD-L1 levels tended to have inferior PFS (HR:1.71; CI:1.00–2.94, *I*^2^ = 82%, *p* = 0.051; Supplementary Fig. 3).

The subgroup analysis of cancer types revealed high pre-treatment sPD-L1 as a strong risk factor in the NSCLC subgroup (HR:2.08; CI:1.81–2.38, *I*^2^ = 0% *p* < 0.001), whereas rather heterogeneous results were observed in RCC (HR:0.67; CI:0.12–3.86, *I*^2^ = 88% *p* = 0.653), melanoma (HR:1.18; CI: 0.56–2.50, *I*^2^ = 74%, *p* = 0.668) and mixed cohorts (HR:0.96; CI:0.47–1.96, *I*^2^ = 74%, *p* = 0.903) (Fig. 3).

### Pre-treatment sPD-1 and PFS and OS

Three articles reported PFS for sPD-1 (HR:1.16; CI:0.23–5.75, *I*^2^ = 89%, *p* = 0.858) (Supplementary Fig. 4) with heterogeneous results. Meyo et al. in NSCLC and Ugurel et al. found in melanoma that higher sPD-1 level patients had shorter PFS, whereas Incorvaia et al. found the opposite result in metastatic RCC [38, 42, 46].

Meyo et al*.* (HR:2.28; CI:1.11–4.68; *p* = 0.025) and Ugurel et al*.* (HR:2.70; CI:1.10–6.25; *p* = 0.055) reported sPD-1 and OS [42, 46].

### sPD-L1 levels strongly increase during anti-PD-L1 therapy

Ten articles reported both pre-treatment and on-treatment sPD-L1 levels in 12 tumor entities. Serum sPD-L1 levels remained unchanged under anti-PD-1 therapy, whereas anti-PD-L1 therapy caused a remarkable (27.67-fold) elevation of sPD-L1 levels (Table 2). Two articles reported both pre-treatment and on-treatment sPD-1 levels during anti-PD-1 (nivolumab) therapy [38, 42] (Table 2).

**Table 2:** Dynamic changes of sPD-L1 and sPD-1 levels before and after 1–3 months of immune checkpoint inhibitor therapy

Author (year)	Type of cancer	Type of treatment	No. of patients	Pre-treatment median sPD-L1 (pg/mL)	No. of patients	On-treatment (1–3 months) median sPD-L1 (pg/mL)	Fold change
Incorvaia et al*.* 2020 [38]	RCC	anti-PD-1	9	1090.0 (R 470.0–2410.0)	9	730.0 (R 560.0–1390.0)	0.67
Meyo et al. 2020 [42]	NSCLC	anti-PD-1	50	160.0 (IQR 30.0–440.0)	50	130.0 (IQR 30.0–380.0)	0.81
Mahoney et al. 2021 [40]	Melanoma	anti-PD-1	78	2312.0	78	2247.0	0.97
Yang et al. 2021 [47]	NSCLC	anti-PD-1	19	37.7 (R 15.6–152.0)	19	36.7 (R 15.6–109.0)	0.97
Mahoney et al*.* 2021 [40]	RCC	anti-PD-1	91	1978.0	91	2179.0	1.10
Costantini et al. 2018 [37]	NSCLC	anti-PD-1	43	39.8 (IQR 29.8–59.2)	43	51.6 (IQR 31.9–72.1)	1.30
Ando et al. 2019 [34]	mixed	anti-PD-1	21	347.4 (R 251.9–1491.1)	9	468.8 (R 256.5–881.3)	1.35
Oh et al. 2021 [45]	Genitourinary	Mixed	10	11.8 (R 5.9–21.5)	10	17.1 (R 6.0–93.5)	1.46
Castello et al. 2020 [35]	NSCLC	anti-PD-1	20	27.2 (R 11.2–61.3)	20	43.9 (R 19.6–77.8)	1.61
Oh et al. 2021 [45]	NSCLC	Mixed	16	15.0 (R 3.8–51.9)	10	58.4 (R 8.7–139.5)	3.89
Krafft et al. 2021 [10]	Urothelial	anti-PD-L1	19	71.2 (R 42.2–192.1)	8	1946.5 (R 1694.0–1993.0)	27.34
Chiarucci et al. 2020 [36]	Mesothelioma	anti-PD-L1	29	70.0 (R 20.0–190.0)	14	1960.0 (R 1330.0–2750.0)	28.00

Author (year)	Type of cancer	Type of treatment	No. of patients	Pre-treatment median sPD-1 (pg/mL)	No. of patients	On-treatment (1–3 months) median sPD-1 (pg/mL)	Fold change
Incorvaia et al*.* 2020 [38]	RCC	anti-PD-1	9	13,250.0 (R 1220.0- 25,000.0)	9	1230.0 (R 1060.0–1930.0)	0.09
Meyo et al. 2020 [42]	NSCLC	anti-PD-1	50	70.0 (IQR 30.0–180.0)	50	70.0 (IQR 30.0–200.0)	1.0

### The assay method does not seem to influence the correlations between sPD-L1 and OS

**Fig. 4:**
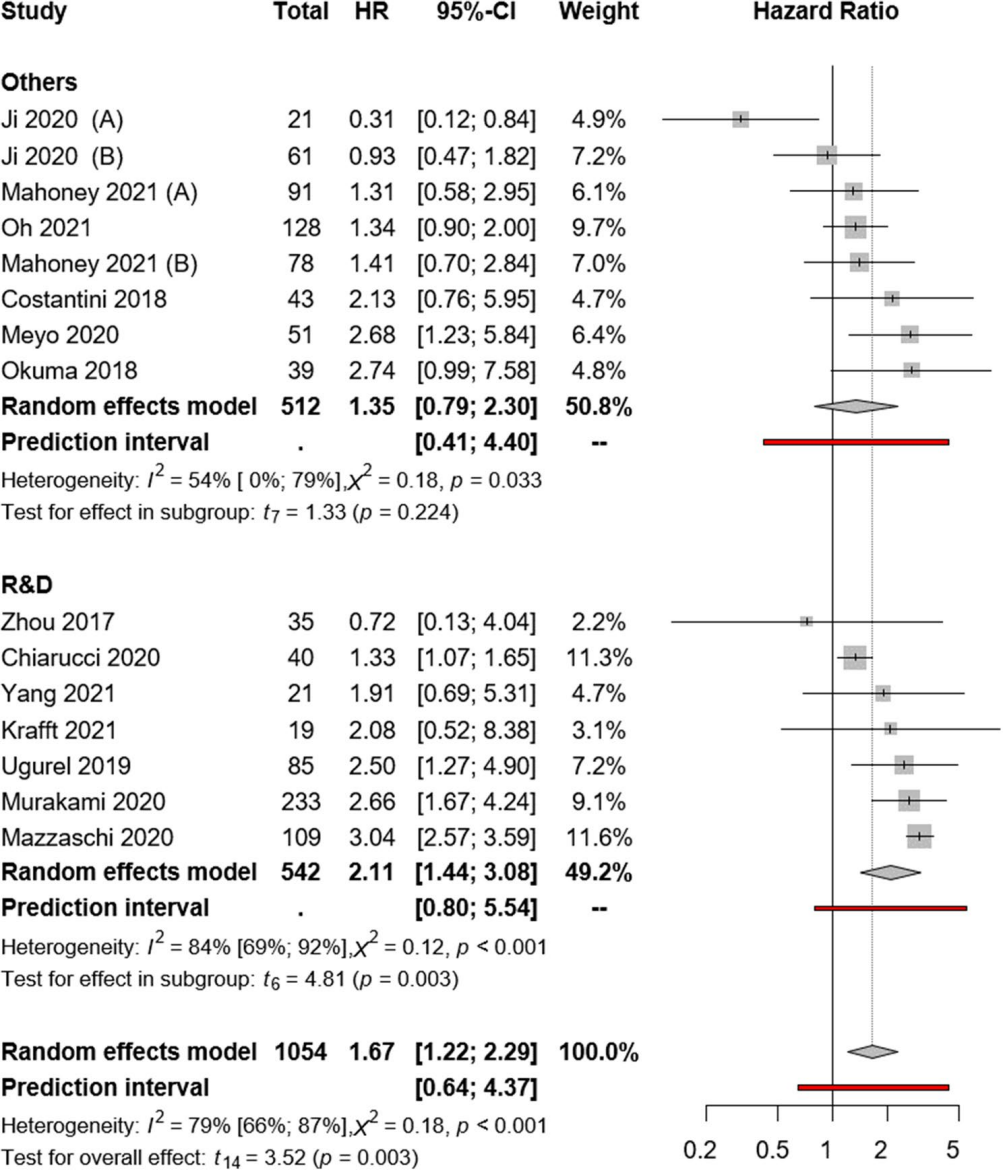
Forest plots representing hazard ratios of OS for sPD-L1 for different ELISA kits

**Fig. 5:**
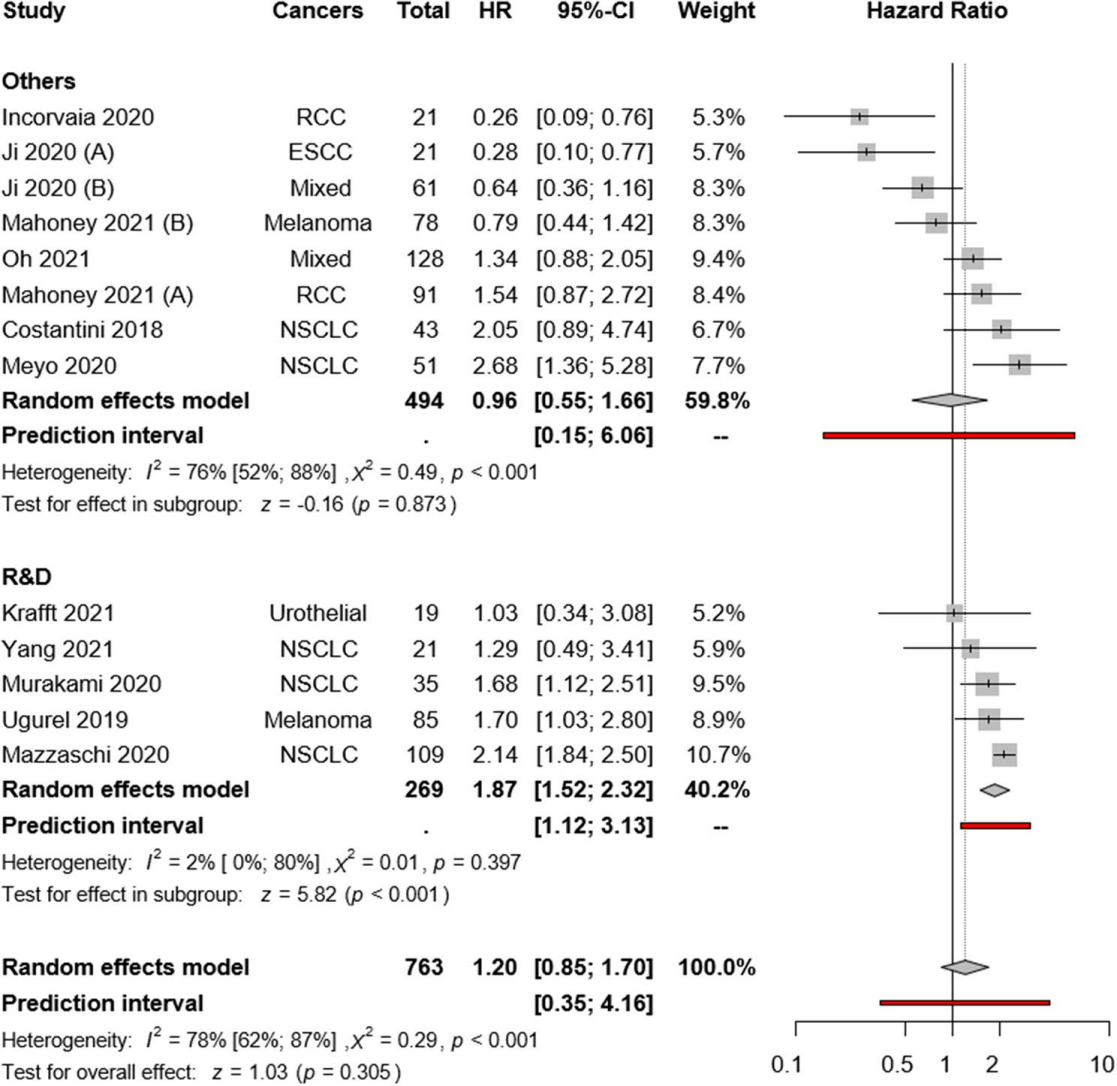
Forest plots representing hazard ratios of PFS for sPD-L1 for different ELISA kits

Our subgroup analysis according to the used assay methods suggested that the sPD-L1 assay method had no major influence on the OS (R&D: HR:2.11; CI:1.44–3.08, *I*^2^ = 84%, *p* = 0.003 vs. “others”: HR:1.35; CI:0.79–2.30, *I*^2^ = 54%, *p* = 0.224; Fig. 4). The same subgroup analysis was further evaluated based on PFS. Our subgroup analysis suggested that the sPD-L1 assay method might influence PFS. (R&D: HR:1.87; CI:1.52–2.32, *I*^2^ = 2%, *p* = 0.025 vs. “others”: HR:0.96; CI:0.55–1.66, *I*^2^ = 76%, *p* = 0.873; Fig. 5). Because of the low number of studies with sPD-1, no comparison was possible between various assay methods.

### Risk of bias assessment and level of evidence

Based on author judgment, 12 out of 16 articles had a low risk of bias, while four carried a moderate risk (Supplementary Fig. 1, Supplementary Table 1).

### Grading

On the basis of GRADEpro™, moderate certainty of the evidence was found for the two primary endpoints (Supplementary Table 2).

## Discussion

This meta-analysis aimed to summarize the data of currently available literature on the prognostic significance of sPD-L1 and sPD-1 in various cancers in the aspect of ICI therapy. Serum sPD-1 and sPD-L1 are easily accessible biomarkers that may help in pre-treatment prognostication and in therapy monitoring of patients who underwent ICI therapy.

In the past few years, several studies assessed the association between sPD-L1 and prognosis in various cancers and treatment settings. Huang et al. constructed a meta-analysis in 2021 to assess the correlation between sPD-L1 and survival in a wide range of human malignancies [49]. The pooled overall estimate showed sPD-L1 as a significant indicator of shorter OS in various cancers. However, the article contained only three ICI therapy-related articles that did not allow to draw firm conclusions. Recently, a significant number of research articles have been published focusing on sPD-L1 (or sPD-1) levels in the context of ICI therapy, and these articles provided contradictory results concerning the prognostic role of sPD-L1. For example, Incorvaia et al*.* found that nivolumab-treated metastatic RCC patients with high sPD-L1, sPD-1, and BTN3A1 levels had better PFS [38]. In contrast, Mahoney et al. in the Checkmate 009 trial found no significant survival benefits for RCC patients with high sPD-L1 levels [40]. In addition, Ji et al. also found significantly higher disease control in ICI-treated ESCC patients as well as better survival rates for patients with high sPD-L1 levels [39]. In contrast, in NSCLC studies, high sPD-L1 levels consequently tended to be associated with shorter patient survival in ICI-treated patients. This finding is in line with the previous meta-analysis by Liao et al*.,* suggesting that low rather than high sPD-L1 levels might have predictive values for ICI treatment [50].

In the present meta-analysis, summarizing data from 16 publications including more than six cancer types and an overall number of 1,054 ICI-treated patients, a 67% higher risk of death was found in patients with high sPD-L1 levels. Similarly, patients with high sPD-L1 levels had a 20% higher risk of disease progression. Interestingly, our subgroup analyses for different tumor entities revealed a heterogeneous pattern. For melanoma, we found three eligible publications with 198 ICI-treated patients, and the observed hazard ratios for OS and PFS revealed a rather heterogeneous picture regarding the prognostic value of sPD-L1 in melanoma. In contrast, for NSCLC, the pooled analysis of six studies with an overall number of 457 ICI-treated patients provided a much more consistent results for both OS and PFS across various studies. Overall, our summary suggests an association between higher sPD-L1 levels and poor prognosis. However, this effect may be different in distinct tumor types. On the basis of these, a tumor type-specific interpretation is suggested for the prognostic value of sPD-L1 in ICI-treated patients. The prognostic value of sPD-L1 is not sufficiently confirmed in melanoma patients, whereas several independent studies confirmed it in NSCLC. Therefore, in NSCLC, pre-treatment sPD-L1 may be a potential biomarker to predict OS and PFS before ICI therapy. On the other hand, in NSCLC, sPD-L1 levels were associated with shorter survival in other therapy settings, suggesting that sPD-L1 might be rather a prognostic than a predictive factor. Further prospective studies are necessary to address this question.

As there are several commercially available sPD-L1 assay kits, we assessed the potential influence of the assay method on study results. Overall, in the 16 included studies, seven different assay kits were applied, with the R&D kit as the most commonly used. However, slightly worse survival rates were found in studies that used the R&D assay, but the visual interpretation of the plot (Figs. 4 and 5) revealed a similar distribution of the articles around the line of no effect. Therefore, we conclude that the ELISA method may not significantly influence the outcomes.

Four articles presented the multivariate analysis of OS and PFS, and the pooled estimate showed high sPD-L1 as an independent risk factor for ICI therapy. However, these articles presented different factors as independent determinators of both OS and PFS. In these four articles, ECOG performance status was consequently found to be a significant independent predictor of survival, whereas tissue expression of PD-L1 was independently associated with poor OS in three articles. Furthermore, two articles showed high neutrophil-to-lymphocyte ratio as an independent predictor of poor OS in a multivariate analysis, suggesting that inflammation status has an inevitable impact on ICI-sensitivity.

Comparison between baseline and on-treatment sPD-L1 levels was possible in 12 studies. Based on our previous observation in urothelial cancer, we hypothesized that anti-PD-L1 therapy leads to an elevation in sPD-L1 levels [10]. Accordingly, in the two studies with presenting patients who received anti-PD-L1 therapy a strong (27- and 28-fold) increase in sPD-L1 levels could be observed [10, 36], whereas no such difference was detected in anti-PD-1-treated patients [34, 35, 37, 38, 40, 42, 45, 47]. Furthermore, sPD-1 levels did not increase after anti-PD-1 (nivolumab) therapy [38, 42]. In contrast, Music et al. observed that sPD-1 elevated after the administration of anti-PD-1 pembrolizumab therapy [51]. Therefore, it appears that anti-PD-L1 rather than anti-PD-1 therapy induces a significant increase in sPD-L1 levels. However, one possible explanation could be that ICIs—especially atezolizumab—can trigger a strong anti-drug-antibody (ADA) production, which may form antibody complexes that can enhance the measured ELISA signal [52]. On the basis of these, the on-treatment flare-up of sPD-L1 seems to be therapy specific for anti-PD-L1 therapy, but the biological and clinical relevance of this elevation needs to be further evaluated.

Our study has some limitations mainly related to the heterogeneity of the included studies regarding their cohort sizes, tumor types, applied ICI drugs, and cut-off values. A further limitation is the unavailability of radiographic response data.

The strength of our study is that it is the first meta-analysis focusing on the prognostic values of sPD-L1 and sPD-1 in a particular group of ICI-treated cancer patients. Furthermore, we evaluated 16 eligible studies with > 1,000 cases using both OS and PFS as endpoints and evaluated results in the context of tumor type, assay method, and marker level changes.

## Conclusion

In conclusion, we found significantly worse OS in ICI-treated cancer patients with high baseline sPD-L1 levels, but this association seems to be tumor type dependent. Therefore, we suggest that sPD-L1 as a pre-treatment prognostic biomarker for ICI therapy, which should be interpreted in a tumor type-specific context. In addition, we found a remarkably strong increase in sPD-L1 during anti-PD-L1 treatment. The biological background and clinical significance of this sPD-L1 flare need to be evaluated in future studies. A further prospectively designed biomarker-based randomized clinical trial is of great need to reveal the therapy predictive role of sPD-L1.

### Supplementary Information

Below is the link to the electronic supplementary material.

Supplementary file1 (DOCX 1600 KB)
