# Supplementary material for: Pre-treatment soluble PD-L1 as a predictor of overall survival for immune checkpoint inhibitor therapy: a systematic review and meta-analysis
Source: Cancer Immunol Immunother. 2022 Nov 16;72(5):1061–73. doi: 10.1007/s00262-022-03328-9 (PMC10110702; doi:10.1007/s00262-022-03328-9)
Supplement: Supplementary file 1 — Supplementary file1 (DOCX 1600 KB) [file 262_2022_3328_MOESM1_ESM.docx]

**Supplementary material**

**Pre-treatment soluble PD-L1 as a predictor of overall survival for immune checkpoint inhibitor therapy,** a systematic review and a meta-analysis

**Authors**

Ádám Széles^1,2^, Tamás Fazekas^1,2^, Szilard Váncsa^2,3,4^, Melinda Váradi^1^, Petra Terézia Kovács^1^, Ulrich Krafft^5^, Viktor Grünwald^5^, Boris Hadaschik^5^, Anita Csizmarik^1^, Péter Hegyi^2,3,4^, Alex Váradi^2^, Péter Nyirády^1^, Tibor Szarvas^1,2,5^

**Affiliations:**

1. Department of Urology, Semmelweis University, Budapest, Hungary
2. Centre for Translational Medicine, Semmelweis University, Budapest, Hungary
3. Institute for Translational Medicine, Szentágothai Research Centre, Medical School, University of Pécs, Pécs, Hungary
4. Division of Pancreatic Diseases, Heart and Vascular Center, Semmelweis University, Budapest, Hungary
5. Department of Urology, University of Duisburg-Essen and German Cancer Consortium (DKTK)-University Hospital Essen, Hufelandstraße 55, D-45147 Essen, Germany

**TABLE OF CONTENT**

**Supplementary Table 1.** PRISMA checklist

**Supplementary Table 2.** Inclusion and exclusion criteria for the included articles

**Supplementary Table 3.** Traffic light plot representing risk of bias assessment

**Supplementary Table 4.** Grading of evidence based on GRADEpro^TM^

**Supplementary Figure 1.** Summary plot of risk of bias assessment

**Supplementary Figure 2.** Forest plots representing multivariate Cox proportional hazard ratios of overall survival (OS) for sPD-L1

**Supplementary Figure 3.** Forest plots representing pooled hazard ratios from multivariate analysis of progression-free survival (PFS) for sPD-L1

**Supplementary Figure 4.** Forest plots representing hazard ratios of progression-free survival for sPD-1

**Supplementary Figure 5.** Funnel plot representing publication bias of overall survival for sPD-L1

**Supplementary Figure 6.** Funnel plot representing publication bias of progression-free survival for sPD-L1

**Supplementary Figure 7.** Egger’s test representing publication bias of overall survival for sPD-L1

**Supplementary Figure 8.** Egger’s test representing publication bias of progression-free survival for sPD-L1

**Supplementary Figure 9.** Leave-one-out analysis for OS outcome

**Supplementary Figure 10.** Leave-one-out analysis for PFS outcome

**Supplementary text 1.** Search key

**Supplementary Table 1.** PRISMA checklist [1]

| **Section and topic** | **Item #** | **Checklist item** | **Location where item is reported** |  |
| --- | --- | --- | --- | --- |
| **Title** | | | | |
| Title | 1 | Identify the report as a systematic review. | 1 |  |
| **Abstract** | | | | |
| Abstract | 2 | See the PRISMA 2020 for Abstracts checklist (table 2). | 3 |  |
| **Introduction** | | | | |
| Rationale | 3 | Describe the rationale for the review in the context of existing knowledge. | 4 |  |
| Objectives | 4 | Provide an explicit statement of the objective(s) or question(s) the review addresses. | 5 |  |
| **Methods** | | | | |
| Eligibility criteria | 5 | Specify the inclusion and exclusion criteria for the review and how studies were grouped for the syntheses. | 6 |  |
| Information sources | 6 | Specify all databases, registers, websites, organisations, reference lists and other sources searched or consulted to identify studies. Specify the date when each source was last searched or consulted. | 6 |  |
| Search strategy | 7 | Present the full search strategies for all databases, registers and websites, including any filters and limits used. | 6 |  |
| Selection process | 8 | Specify the methods used to decide whether a study met the inclusion criteria of the review, including how many reviewers screened each record and each report retrieved, whether they worked independently, and if applicable, details of automation tools used in the process. | 6 |  |
| Data collection process | 9 | Specify the methods used to collect data from reports, including how many reviewers collected data from each report, whether they worked independently, any processes for obtaining or confirming data from study investigators, and if applicable, details of automation tools used in the process. | 7 |  |
| Data items | 10a | List and define all outcomes for which data were sought. Specify whether all results that were compatible with each outcome domain in each study were sought (e.g. for all measures, time points, analyses), and if not, the methods used to decide which results to collect. | 7 |  |
|  | 10b | List and define all other variables for which data were sought (e.g. participant and intervention characteristics, funding sources). Describe any assumptions made about any missing or unclear information. | 7 |  |
| Study risk of bias assessment | 11 | Specify the methods used to assess risk of bias in the included studies, including details of the tool(s) used, how many reviewers assessed each study and whether they worked independently, and if applicable, details of automation tools used in the process. | 7 |  |
| Effect measures | 12 | Specify for each outcome the effect measure(s) (e.g. risk ratio, mean difference) used in the synthesis or presentation of results. | 7 |  |
| Synthesis methods | 13a | Describe the processes used to decide which studies were eligible for each synthesis (e.g. tabulating the study intervention characteristics and comparing against the planned groups for each synthesis (item #5)). | 7 |  |
|  | 13b | Describe any methods required to prepare the data for presentation or synthesis, such as handling of missing summary statistics, or data conversions. | 7 |  |
|  | 13c | Describe any methods used to tabulate or visually display results of individual studies and syntheses. | 7 |  |
|  | 13d | Describe any methods used to synthesise results and provide a rationale for the choice(s). If meta-analysis was performed, describe the model(s), method(s) to identify the presence and extent of statistical heterogeneity, and software package(s) used. | 7 |  |
|  | 13e | Describe any methods used to explore possible causes of heterogeneity among study results (e.g. subgroup analysis, meta-regression). | 7 |  |
|  | 13f | Describe any sensitivity analyses conducted to assess robustness of the synthesised results. | 7 |  |
| Reporting bias assessment | 14 | Describe any methods used to assess risk of bias due to missing results in a synthesis (arising from reporting biases). | 7 |  |
| Certainty assessment | 15 | Describe any methods used to assess certainty (or confidence) in the body of evidence for an outcome. | 7 |  |
| **Results** | | | | |
| Study selection | 16a | Describe the results of the search and selection process, from the number of records identified in the search to the number of studies included in the review, ideally using a flow diagram (see fig 1). | 8 |  |
|  | 16b | Cite studies that might appear to meet the inclusion criteria, but which were excluded, and explain why they were excluded. | 8 |  |
| Study characteristics | 17 | Cite each included study and present its characteristics. | 8 |  |
| Risk of bias in studies | 18 | Present assessments of risk of bias for each included study. | Suppl. |  |
| Results of individual studies | 19 | For all outcomes, present, for each study: (a) summary statistics for each group (where appropriate) and (b) an effect estimate and its precision (e.g. confidence/credible interval), ideally using structured tables or plots. | Figures |  |
| Results of syntheses | 20a | For each synthesis, briefly summarise the characteristics and risk of bias among contributing studies. | Suppl. |  |
|  | 20b | Present results of all statistical syntheses conducted. If meta-analysis was done, present for each the summary estimate and its precision (e.g. confidence/credible interval) and measures of statistical heterogeneity. If comparing groups, describe the direction of the effect. | 8-10 |  |
|  | 20c | Present results of all investigations of possible causes of heterogeneity among study results. | 8-10 |  |
|  | 20d | Present results of all sensitivity analyses conducted to assess the robustness of the synthesised results. | 8-10 |  |
| Reporting biases | 21 | Present assessments of risk of bias due to missing results (arising from reporting biases) for each synthesis assessed. | 8-10 |  |
| Certainty of evidence | 22 | Present assessments of certainty (or confidence) in the body of evidence for each outcome assessed. | 8-10 |  |
| **Discussion** | | | | |
| Discussion | 23a | Provide a general interpretation of the results in the context of other evidence. | 11 |  |
|  | 23b | Discuss any limitations of the evidence included in the review. | 11-13 |  |
|  | 23c | Discuss any limitations of the review processes used. | 11-13 |  |
|  | 23d | Discuss implications of the results for practice, policy, and future research. | 11-13 |  |
| **Other information** | | | | |
| Registration and protocol | 24a | Provide registration information for the review, including register name and registration number, or state that the review was not registered. | 6 |  |
|  | 24b | Indicate where the review protocol can be accessed, or state that a protocol was not prepared. | 6 |  |
|  | 24c | Describe and explain any amendments to information provided at registration or in the protocol. | 6 |  |
| Support | 25 | Describe sources of financial or non-financial support for the review, and the role of the funders or sponsors in the review. | 2 |  |
| Competing interests | 26 | Declare any competing interests of review authors. | 2 |  |
| Availability of data, code, and other materials | 27 | Report which of the following are publicly available and where they can be found: template data collection forms; data extracted from included studies; data used for all analyses; analytic code; any other materials used in the review. |  |  |

**Supplementary Table 2.** Inclusion and exclusion criteria for the included articles

| **Article** | **Inclusion criteria** | **Exclusion criteria** |
| --- | --- | --- |
| Ando *et al.* 2019 [2] | “We retrospectively analyzed data for 21 patients, 11 with first-line or previously treated NSCLC, nine with gastric cancer and one with bladder cancer, who received ICI treatment (240 mg nivolumab intravenously every 2 weeks, or 200 mg pembrolizumab intravenously every 3 weeks) at Showa University Hospital from January 2017 to April 2019.” | No information |
| Castello *et al*. 2020 [3] | “Between July 2017 and May 2019, we prospectively enrolled 20 patients (13 males, 7 females, mean age 72 years) with NSCLC candidate to ICI therapy at our Institution Humanitas Clinical and Research Center. We included in our analysis patients who had serum frozen samples and 18F-FDG PET/CT available, both at baseline and at the first restaging after approximately three or four cycles of ICI. Nivolumab was administered intravenously every two weeks at dosage of 3 mg/kg, while pembrolizumab every three weeks at 200 mg” | No information |
| Chiarucci *et al*. 2020 [4] | “A number of 121 patients diagnosed with tumors of different histotypes and treated with anti-PD-L1, anti-PD-1, or anti-CTLA-4 mAbs were investigated They included 40 mesothelioma patients enrolled in the NIBIT-MESO-1 trial (anti-CTLA-4 mAb tremelimumab combined with the anti-PD-L1 mAb durvalumab) and other 32 solid cancer patients treated with the anti-PD-L1 mAbs atezolizumab or durvalumab alone or in combination with the anti-CTLA-4 mAb tremelimumab; 20 solid cancer patients treated with the anti-CTLA-4 mAbs ipilimumab, or tremelimumab; and 29 solid cancer patients treated with the anti-PD-1 mAbs pembrolizumab, or nivolumab. Twenty-two healthy volunteers were used as a control cohort after signing an informed consent form.” | No information |
| Costantini *et al*. 2018 [5] | “This study was an exploratory study, based on the analysis of consecutive patients prospectively included in the Department of Respiratory Medicine and Thoracic Oncology (APHP – Ambroise Pare Hospital) between July 2015 and September 2017. Exploratory endpoints were ORR, clinical benefit (i.e. complete response, partial response or stability, according to iRECIST, lasting 6 months or more after initiation of nivolumab treatment), PFS, OS, grade 3 – 4 toxicity (according to CTCAE v4.0), according to plasmatic concentrations of various circulating biomarkers.” | No information |
| Incorvaia *et al.* 2020 [6] | “We performed a prospective study including a cohort of 56 patients with histologically confirmed diagnosis of ccRCC, 36 patients from University Hospital Policlinico “P. Giaccone” of Palermo (Italy) and a validation cohort of 20 patients from I.R.C. C.S. San Matteo University Hospital Foundation of Pavia (Italy). Peripheral blood samples from ccRCC patients were prospectively obtained from March 2017 to January 2019. The study population included patients with advanced disease candidate to the anti-PD-1 nivolumab as second line treatment, based on medical choice and current therapeutic options.” | No information |
| Ji *et al*. 2020 [7] | “The two patient cohorts involved in this study were available from 2 clinical trials (NCT03101488, NCT02937116) conducted in the Department of GI Oncology, The Fifth Medical Center, Chinese PLA General Hospital between November 2016 and August 2018. Only patients with Eastern Cooperative Oncology Group (ECOG) performance status of 0 or 1 were included. All patients who failed or did not tolerate standard treatments (In the discovery cohort, ESCC patients who failed or did not tolerate first-line chemotherapy; in the validated cohort, all patients who failed or did not tolerate at least first-line standard treatment) were treated with anti-PD-1/PD-L1 monotherapy,” | “while the patients who treated with anti-PD-1/PD-L1 antibody combination with other therapies were excluded.” |
| Krafft *et al*. 2021 [8] | “In addition, pretreatment serum samples were drawn from 12 urothelial cancer patients (10 males, 2 females), who received ICI therapy (atezolizumab (n = 11) and pembrolizumab (n = 1)) either in 2nd line setting (n = 9) or in case of platinum-ineligibility (n = 3). Samples were collected between 4/2019 and 3/2020 at the Department of Urology, Semmelweis University. The vast majority (11/12) of ICI treatments were applied in the second line setting and one patient received neoadjuvant ICI therapy” | No information |
| Mahoney *et al*. (Melanoma cohort) 2021 [9] | “Inclusion Criteria:  Men and women >18 years   - Eastern Cooperative Oncology Group (ECOG) status = 0 to 1 - Subjects with unresectable Stage III or IV melanoma who are either refractory or intolerant to, or have refused standard therapy for treatment of metastatic melanoma - Subject must have histologic or cytologic confirmation of advanced melanoma - Subjects must have at least one measurable lesion at baseline by computed tomography (CT) or magnetic resonance imaging (MRI) as per Response Evaluation Criteria in Solid Tumors (RECIST) 1.1 criteria - Subjects must have at least 1 tumor site that can be biopsied at acceptable clinical risk and must consent to pre- and post-treatment biopsies” | “Exclusion Criteria:   - Active or progressing brain metastases - Other concomitant malignancies (with some exceptions per protocol) - Active or history of autoimmune disease - Positive test for human immunodeficiency virus (HIV) 1&2 or known acquired immunodeficiency syndrome (AIDS) - History of any hepatitis Prior therapy with any antibody/drug that targets the T cell coregulatory proteins, including but not limited to, anti-PD-1, anti-PD-L1, anti-PD-L2, anti-CD137, anti-OX-40,and anti-CD40 antibodies. However, half the patients must have progressed on anti Cytotoxic T lymphocyte-associated antigen 4 (anti-CTLA4) monoclonal antibody therapy” |
| Mahoney *et al*. (RCC cohort) 2021 [9] | “Eligible patients had histologically confirmed mRCC with a clear-cell component, measurable disease defined by Response Evaluation Criteria in Solid Tumors (RECIST) v1.1, Karnofsky performance score of ≥70%, presence of soft-tissue tumor lesions that could be biopsied at baseline and on treatment, and adequate organ and marrow function. To be eligible for the previously treated groups, patients must have been treated with between 1 and 3 previous systemic therapies for RCC, with progression following the most recent therapy within 6 months of study enrollment. For the treatment-naïve group, patients must not have received any previous systemic therapy in the metastatic or adjuvant setting” | “Exclusion criteria included active central nervous system metastases within 30 days of study enrollment; active or prior autoimmune disease; prior malignancy unless complete remission occurred ≥2 years prior to study enrollment; and previous treatment with anti–CTLA-4, anti–PD-1, anti–PD-L1, anti–PD-L2, anti-CD137, anti-CD40, or anti-OX40 antibodies” |
| Mazzaschi *et al.* 2020 [10] | “patients histologically or cytologically diagnosed with locally advanced or metastatic NSCLC disease (stage IIIB or IV), not susceptible to curative therapy; eligibility for ICIs monotherapy (e.g. no active autoimmune disease preventing its administration, steroid intake ≤ 10 mg/die of prednisone); no previous treatment with ICIs.” | No information |
| Meyo *et al*. 2020 [11] | “Inclusion criteria were the administration of at least two cycles of nivolumab, availability of plasma samples at baseline (less than two weeks before the first injection of nivolumab) and at day 28 just before the third infusion. Exclusion criteria were small-cell cancer or a mixed tumor with a neuroendocrine small-cell component.” | No information |
| Murakami *et al.* 2020 [12] | “Patients with advanced or recurrent NSCLC who received nivolumab or pembrolizumab as a first-line to third-line treatment between 1 December 2015, and 31 March 2018, at the National Cancer Center Hospital (Tokyo, Japan) were eligible for inclusion in this study” | “Patients were excluded if they had insufficient serum samples available from before the start of treatment with anti-PD-1 antibody.” |
| Okuma *et al*. 2018 [13] | “Blood samples at baseline from patients treated with nivolumab for NSCLC were prospectively obtained. The clinical information was collected from the patients at the Division of Respiratory Diseases, Department of Internal Medicine, Jikei University School of Medicine and Department of Thoracic Oncology and Respiratory Medicine, Tokyo Metropolitan Cancer and Infectious Diseases Center Komagome Hospital from May 2016 to April 2017 as part of the exploratory investigation. The patients were treated with nivolumab at the dose of 3 mg/kg every 2 weeks until disease progression or unacceptable toxicities in the clinical setting” | No information |
| So *et al*. 2021 [14] | “Patients were eligible for the study if they (1) were 18 years of age or older, (2) had a histologically confirmed malignancy, (3) received ICI treatment at Seoul National University Hospital, (4) had study samples taken before and/or after ICI treatment, and (5) completed a written consent form for research using human derivatives, which allowed for secondary utilization of samples (IRB No. 1104- 086-359).” | “A patient was excluded from the study if they had a diagnosis of two or more types of malignancy within the previous 5 years, withdrew consent before or during the study, or not enough samples were stored for analysis.” |
| Ugurel *et al.* 2019 [15] | “For the two discovery cohorts, patients presenting at the Department of Dermatology, University Hospital Essen, were retrospectively selected according to the following criteria: unresectable metastatic melanoma stage III or IV based on American Joint Committee on Cancer (AJCC, version 7) staging15; first systemic therapy with PD-1 inhibitors as monotherapy or combined with other checkpoint inhibitors (discovery cohort 1), or first systemic therapy with BRAF inhibitors as monotherapy or combined with mitogen-activated protein kinase kinase (MEK) inhibitors (discovery cohort 2); complete documentation of therapy outcome including best overall response (BOR) and progression-free (PFS) and overall (OS) survival.” | No information |
| Yang *et al*. 2021 [16] | “Inclusion Criteria:   1. Pathological histology and/or cytology confirmed non-small lung cancer; 2. Voluntarily enrolled to participate in,better compliance, cooperate with experimental observations, and sign informed consent.” | “Exclusion Criteria:   1. Vital organs (e.g., heart, liver, kidney) have serious dysfunction; 2. Patients with a history of autoimmune disease; 3. Patients with participating in other clinical trials at the same time; 4. Other cases that researchers believe that patients should not participate in the present trial.” |
| Zhou *et al*. 2017 [17] | No information | No information |

**Supplementary Table 3.** Traffic light plot representing risk of bias assessment


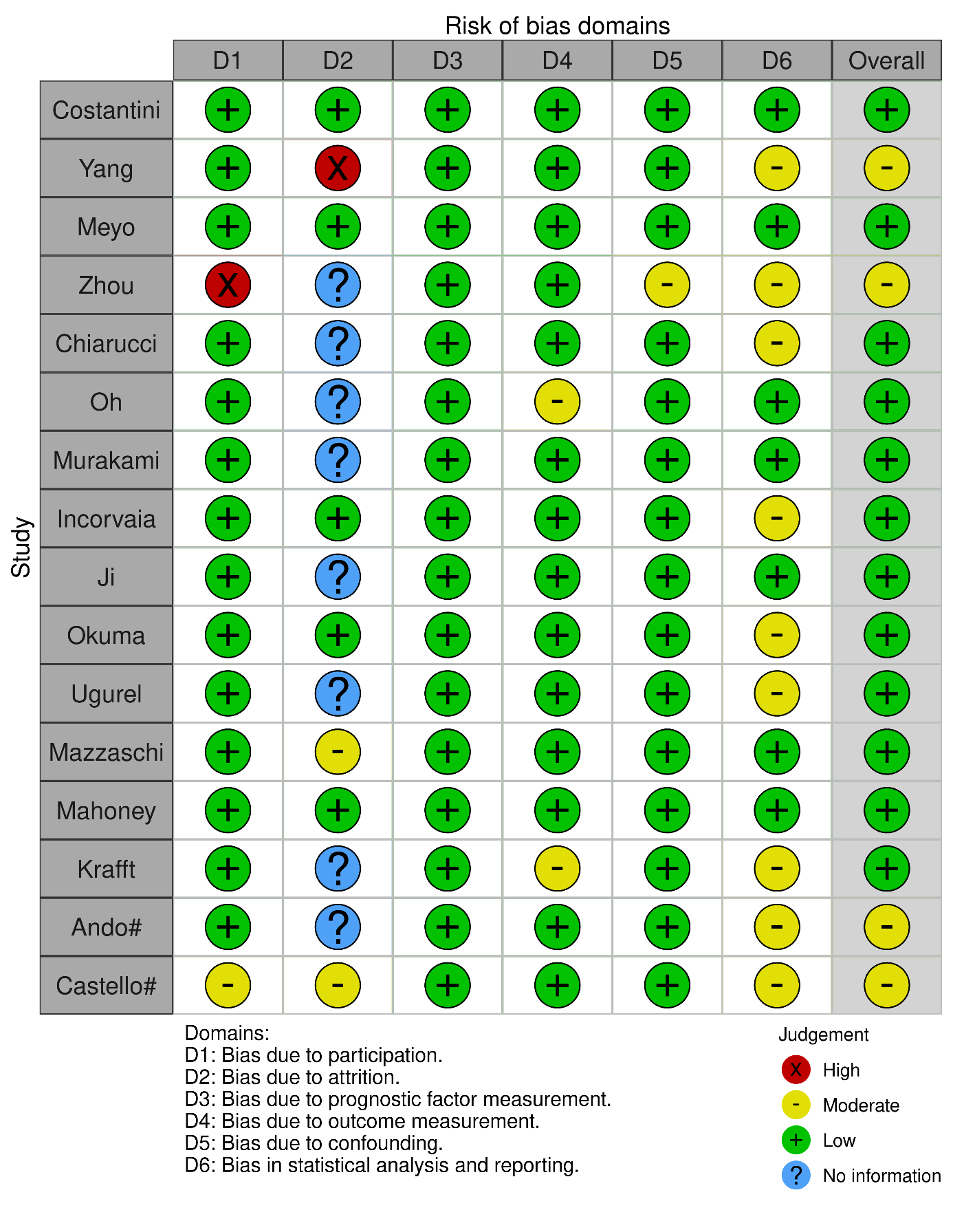


**Supplementary Table 4.** Grading of evidence based on GRADEpro^TM^

| **Certainty assessment** | | | | | | | **№ of patients** | | **Effect** | | **Certainty** | **Importance** |
| --- | --- | --- | --- | --- | --- | --- | --- | --- | --- | --- | --- | --- |
| **№ of studies** | **Study design** | **Risk of bias** | **Inconsistency** | **Indirectness** | **Imprecision** | **Other considerations** | **high sPD-L1 levels** | **low sPD-L1 levels** | **Relative (95% CI)** | **Absolute (95% CI)** |  |  |
| **New outcome (assessed with: HR)** | | | | | | | | | | | | |
| 15 | observational studies | not serious | not serious | not serious | not serious | none |  |  | **HR 1.67** (1.26 to 2.23) [New outcome] | **-- per 1 000** (from -- to --) | ⨁⨁⨁◯ Moderate | IMPORTANT |
|  |  |  |  |  |  |  | - | 0.0% |  | **-- per 1 000** (from -- to --) |  |  |
| **New outcome (assessed with: HR)** | | | | | | | | | | | | |
| 12 | observational studies | not serious | not serious | not serious | not serious | publication bias strongly suspected^a^ |  |  | **HR 1.20** (0.85 to 1.70) [New outcome] | **-- per 1 000** (from -- to --) | ⨁⨁⨁◯ Moderate | IMPORTANT |
|  |  |  |  |  |  |  | - | 0.0% |  | **-- per 1 000** (from -- to --) |  |  |

**CI:** confidence interval; **HR:** hazard Ratio

#### Explanations

a. Eggers’ Test and funnel plot shows publication bias (t = -3.32; df = 11; p = 0.007), BUT! the meta-analysis contains few studies.


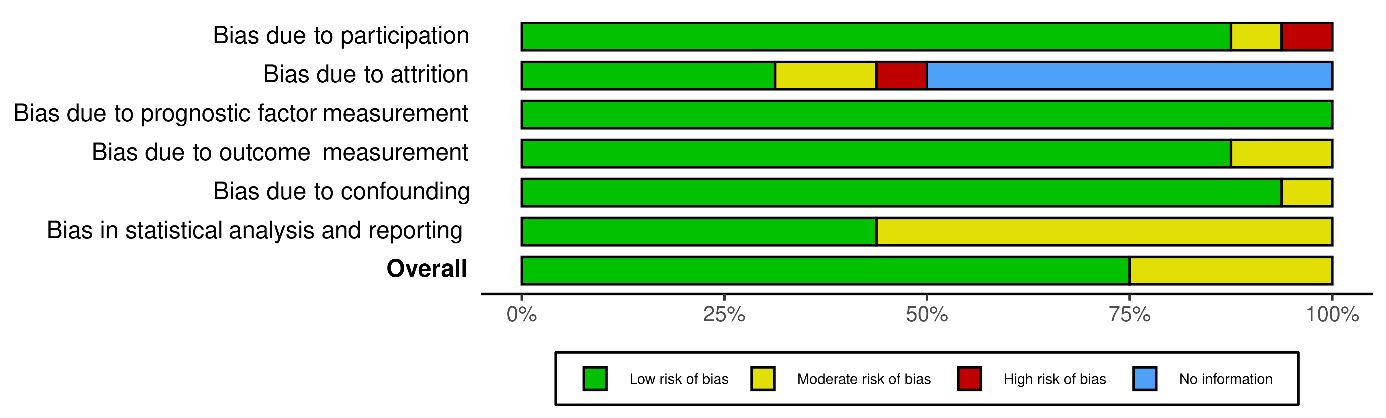
**Supplementary Figure 1.** Summary plot of risk of bias assessment


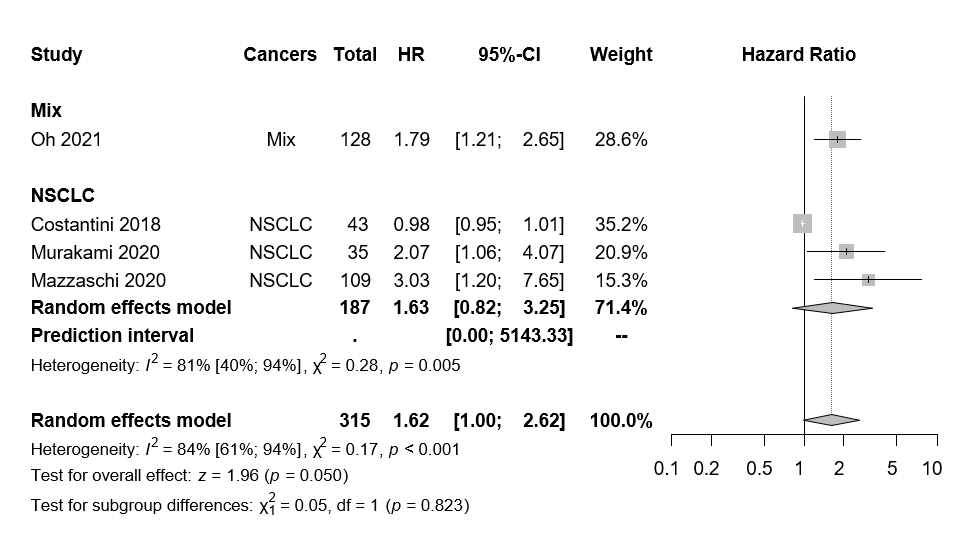
**Supplementary Figure 2.** Forest plots representing multivariate Cox proportional hazard ratios of overall survival (OS) for sPD-L1


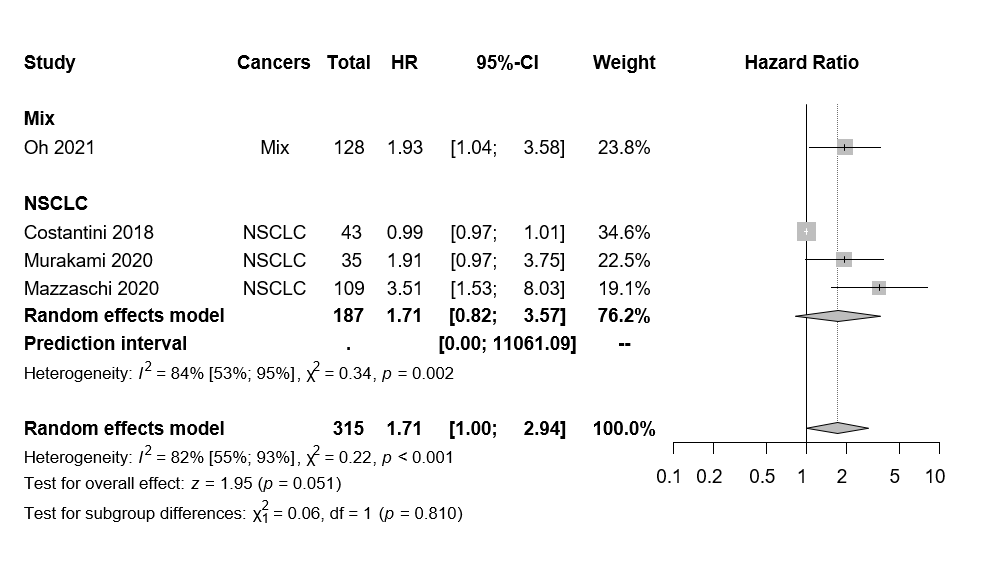
**Supplementary Figure 3.** Forest plots representing pooled hazard ratios from multivariate analysis of progression-free survival (PFS) for sPD-L1


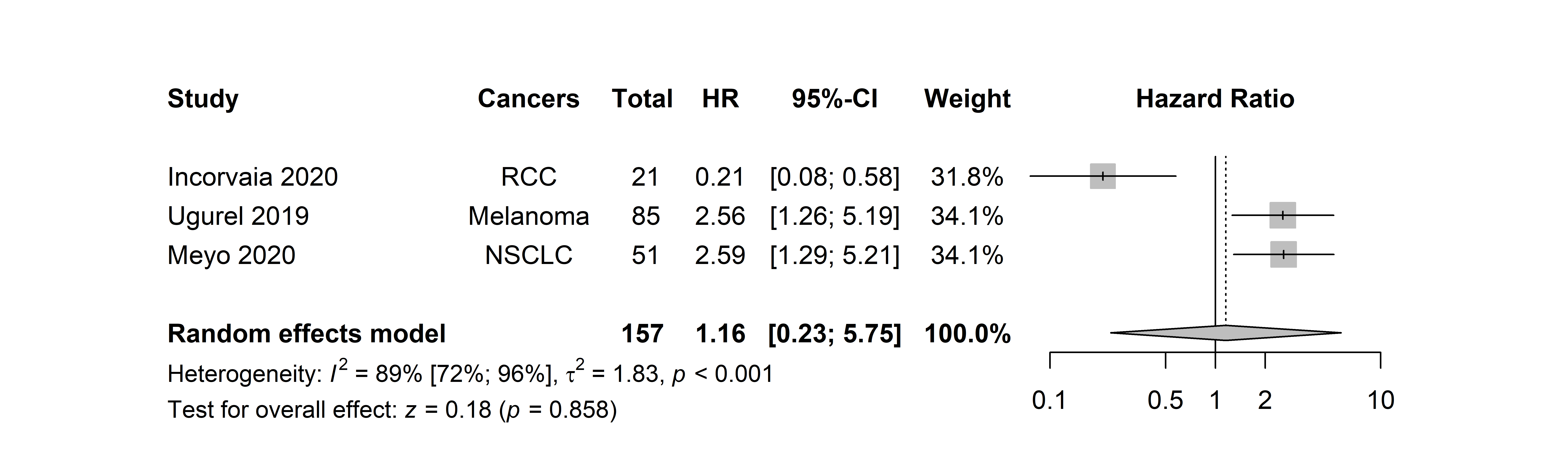
**Supplementary Figure 4.** Forest plots representing hazard ratios of progression-free survival for sPD-1


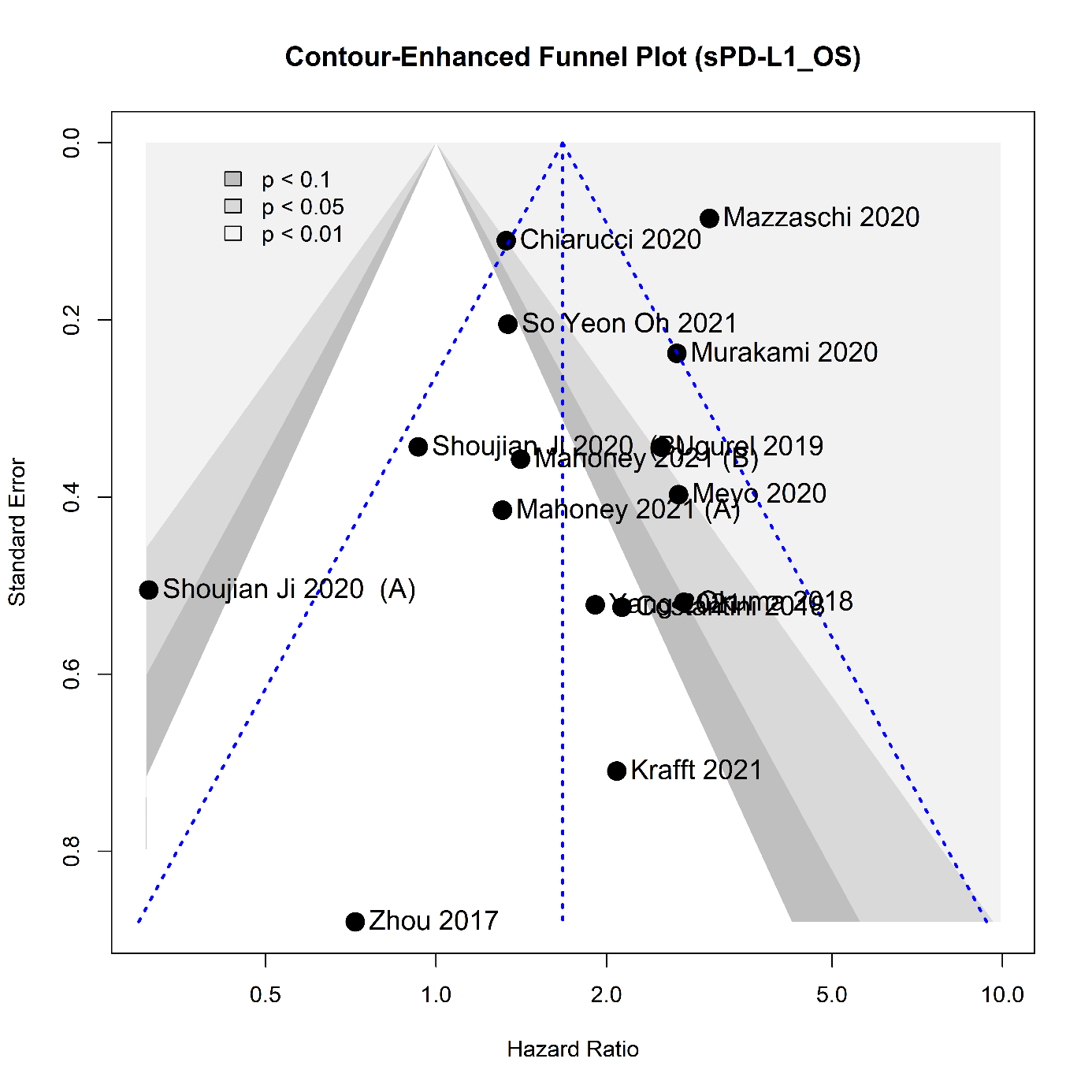
**Supplementary Figure 5.** Funnel plot representing publication bias of overall survival for sPD-L1


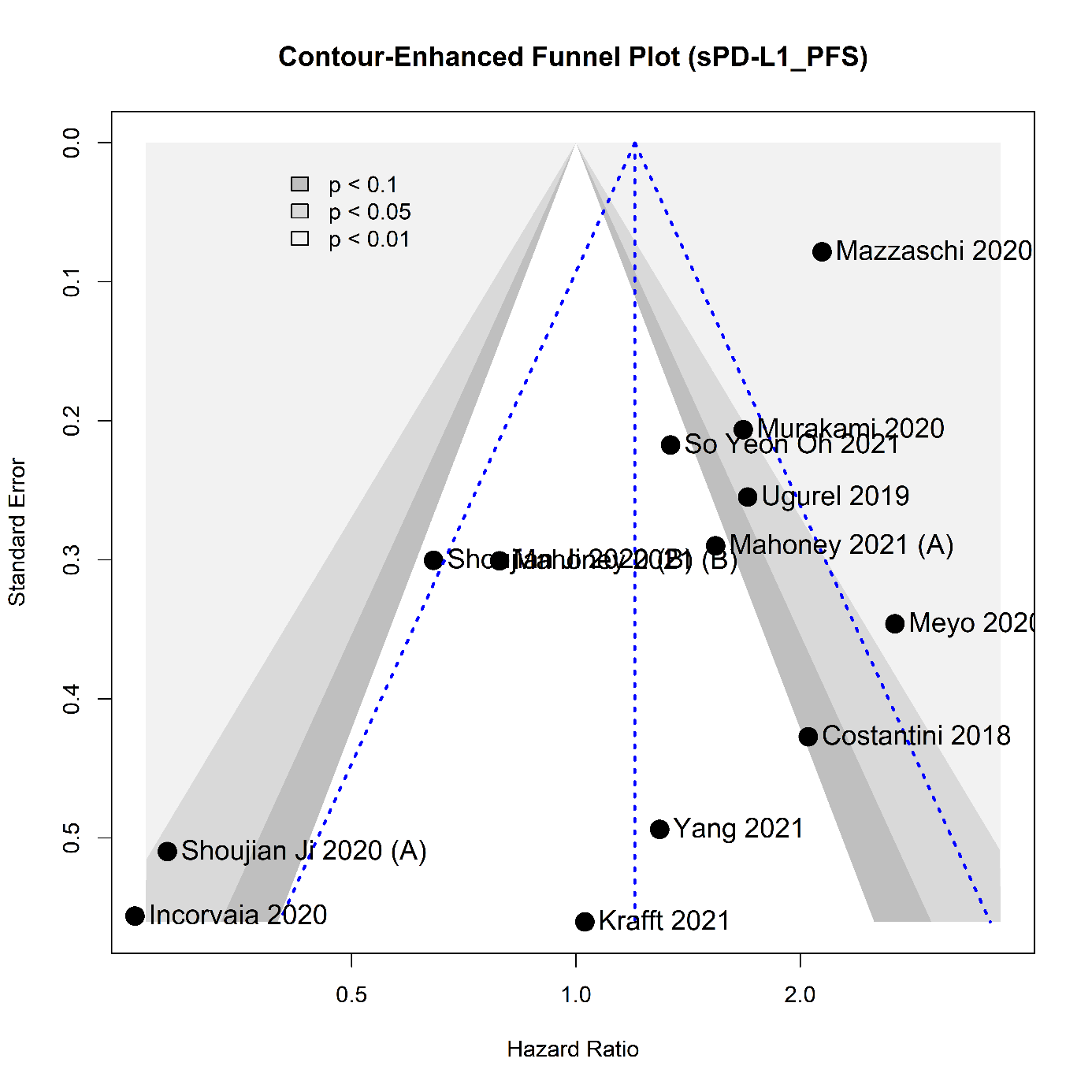
**Supplementary Figure 6.** Funnel plot representing publication bias of progression-free survival for sPD-L1


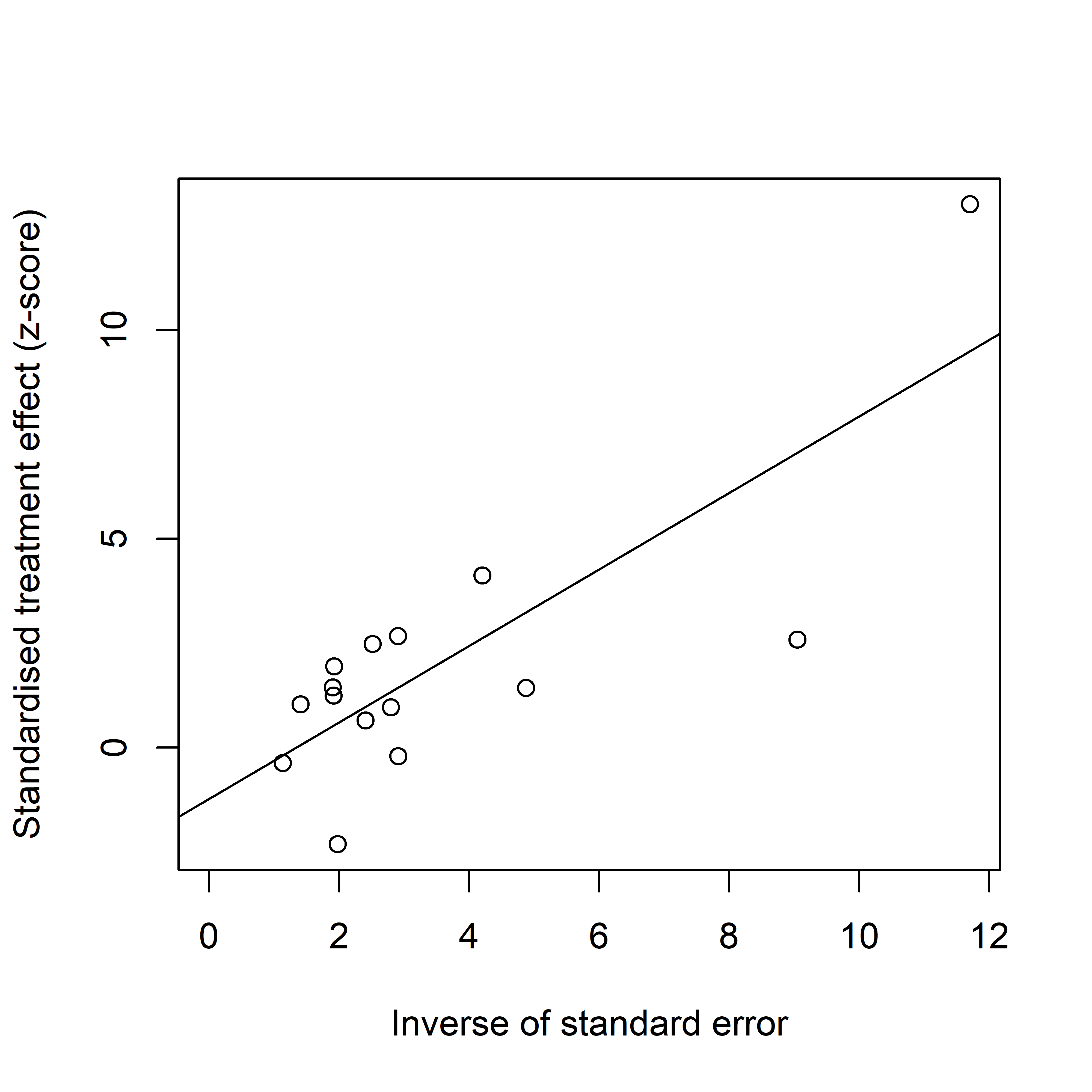


**Supplementary Figure 7.** Egger’s test representing publication bias of overall survival for sPD-L1


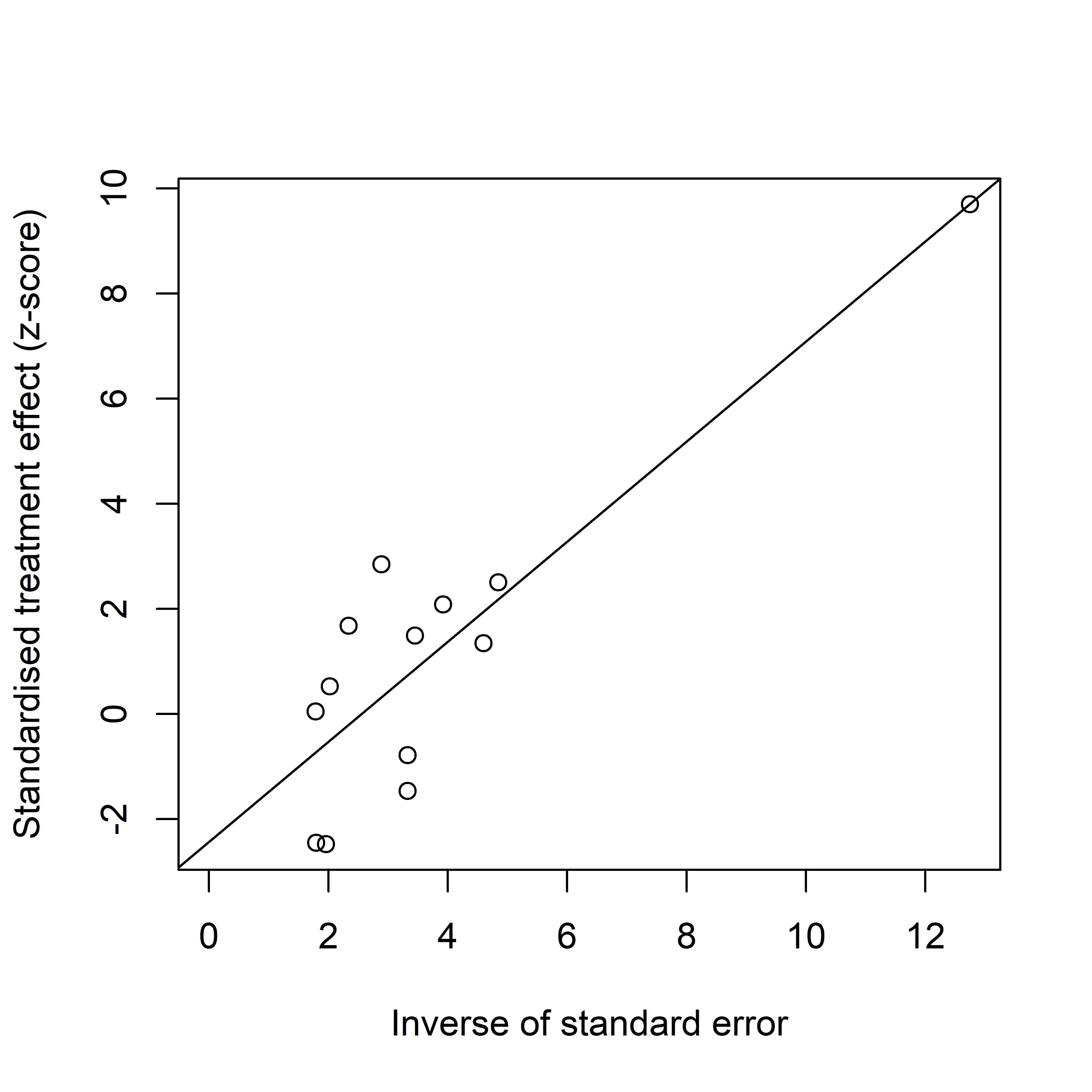


**Supplementary Figure 8.** Egger’s test representing publication bias of progression-free survival for sPD-L1


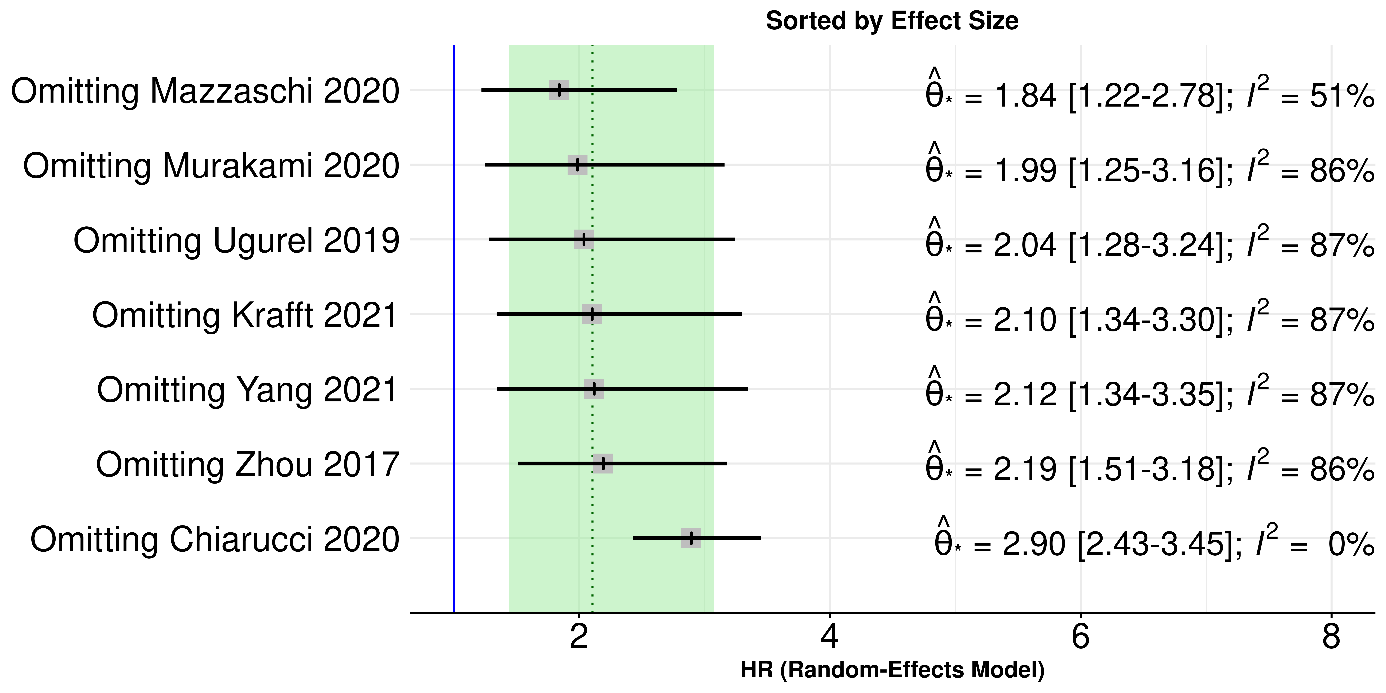


“R&D” Subgroup


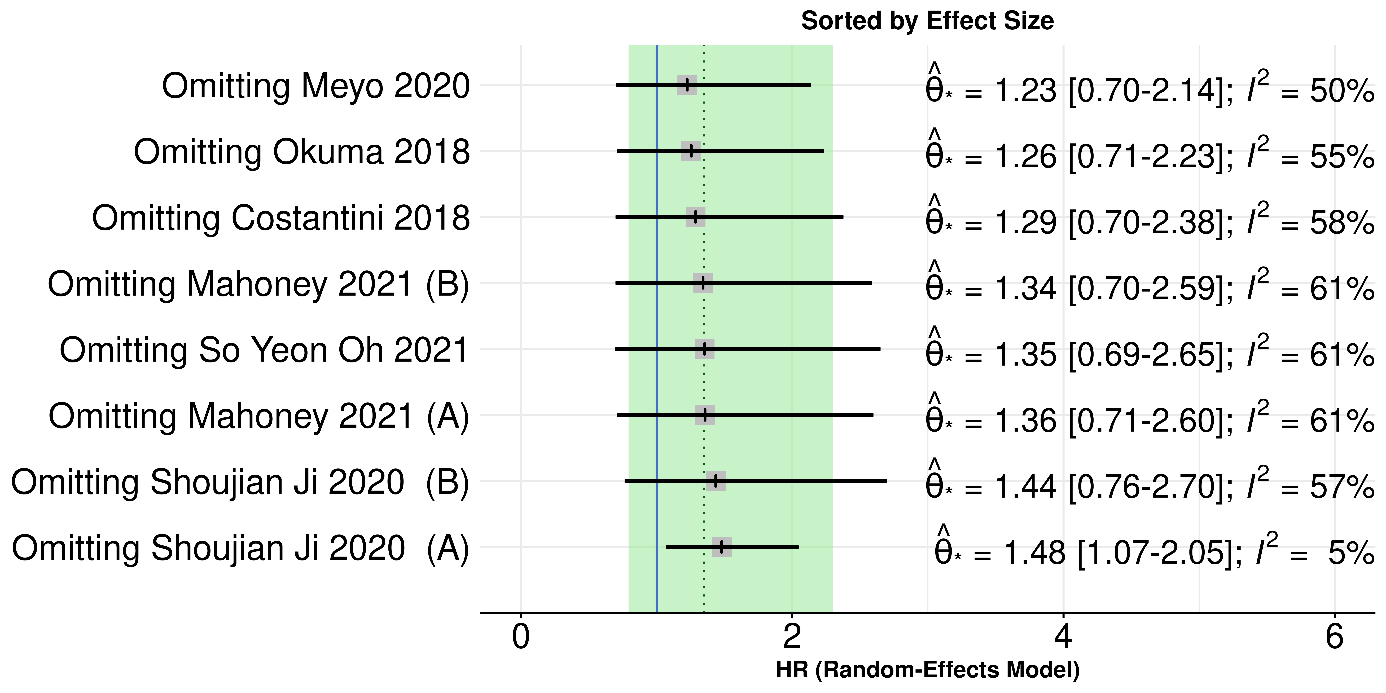


“Others” Subgroup

**Supplementary Figure 9.** Leave-one-out analysis for OS outcome


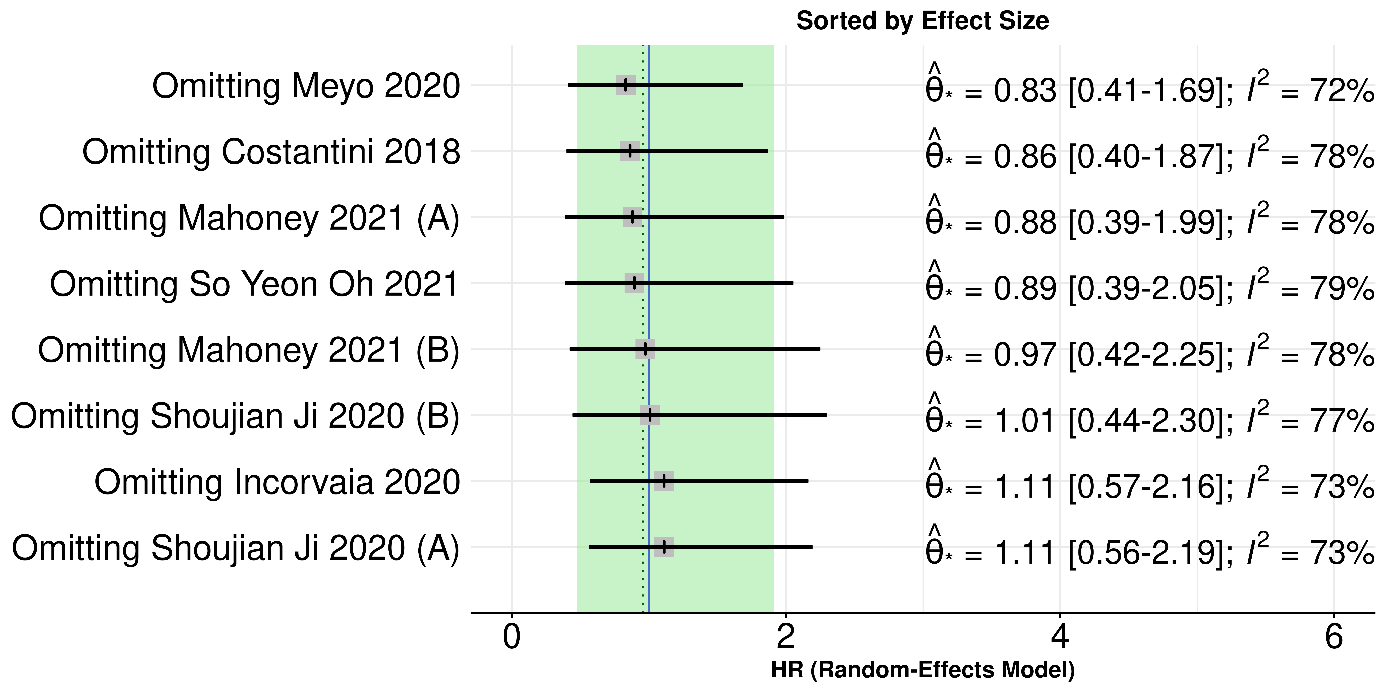


“Others” Subgroup
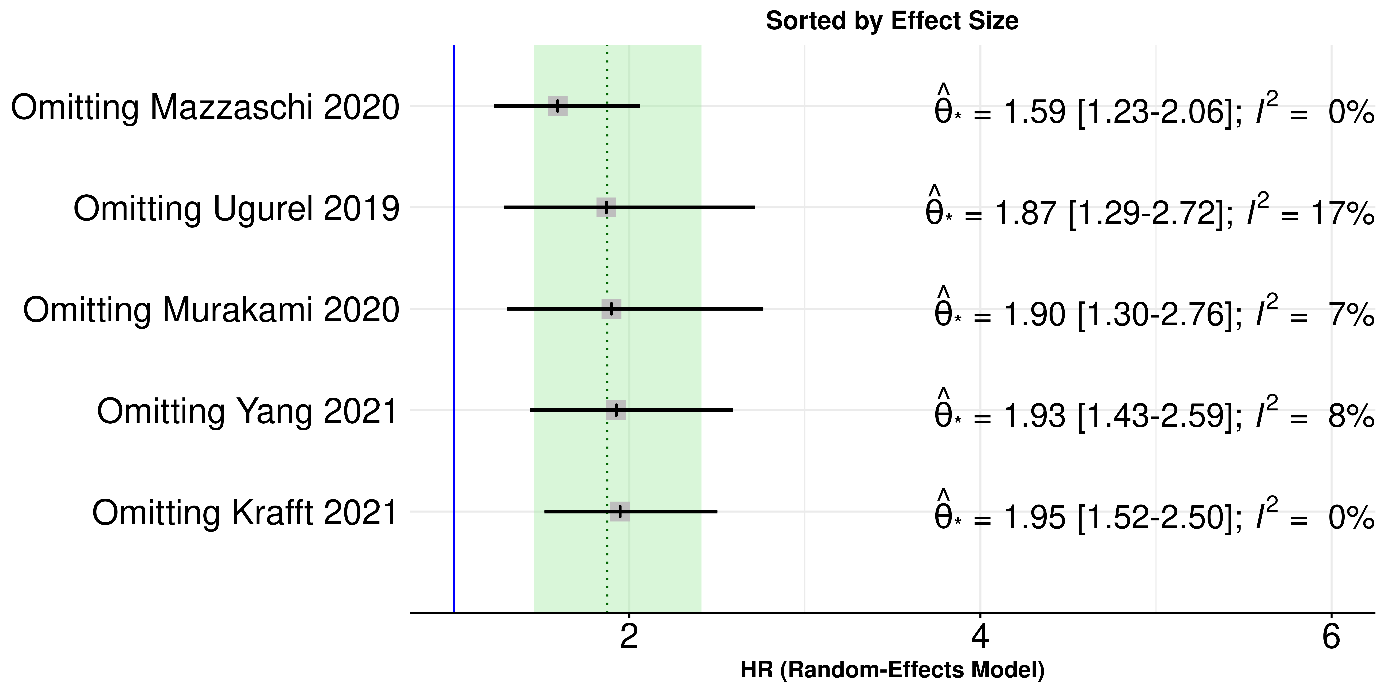


“R&D” Subgroup

**Supplementary Figure 10.** Leave-one-out analysis for PFS outcome

**Supplementary text 1.** Search key

The following search key was used:

(sPD-1 OR “serum PD-1” OR “soluble PD-1” OR “plasma PD-1” OR sPD-L1 OR “serum PD-L1” OR “soluble PD-L1” OR “plasma PD-L1” OR “blood PD-L1”) and (durvalumab OR Imfinzi OR pembrolizumab OR Keytruda OR nivolumab OR Opdivo OR atezolizumab OR Tecentriq OR avelumab OR Bavencio OR Cemiplimab OR Libtayo OR immune checkpoint inhibitor OR PD-1 inhibitor OR PD-L1 inhibitor).

**REFERENCES**

1. Page, M.J.; McKenzie, J.E.; Bossuyt, P.M.; Boutron, I.; Hoffmann, T.C.; Mulrow, C.D.; Shamseer, L.; Tetzlaff, J.M.; Akl, E.A.; Brennan, S.E.; et al. The PRISMA 2020 statement: an updated guideline for reporting systematic reviews. *BMJ* **2021**, *372*, n71, doi:10.1136/bmj.n71.

2. Ando, K.; Hamada, K.; Watanabe, M.; Ohkuma, R.; Shida, M.; Onoue, R.; Kubota, Y.; Matsui, H.; Ishiguro, T.; Hirasawa, Y.; et al. Plasma Levels of Soluble PD-L1 Correlate With Tumor Regression in Patients With Lung and Gastric Cancer Treated With Immune Checkpoint Inhibitors. *Anticancer Res* **2019**, *39*, 5195-5201, doi:10.21873/anticanres.13716.

3. Castello, A.; Rossi, S.; Toschi, L.; Mansi, L.; Lopci, E. Soluble PD-L1 in NSCLC Patients Treated with Checkpoint Inhibitors and Its Correlation with Metabolic Parameters. *Cancers (Basel)* **2020**, *12*, doi:10.3390/cancers12061373.

4. Chiarucci, C.; Cannito, S.; Daffina, M.G.; Amato, G.; Giacobini, G.; Cutaia, O.; Lofiego, M.F.; Fazio, C.; Giannarelli, D.; Danielli, R.; et al. Circulating Levels of PD-L1 in Mesothelioma Patients from the NIBIT-MESO-1 Study: Correlation with Survival. *Cancers (Basel)* **2020**, *12*, doi:10.3390/cancers12020361.

5. Costantini, A.; Julie, C.; Dumenil, C.; Helias-Rodzewicz, Z.; Tisserand, J.; Dumoulin, J.; Giraud, V.; Labrune, S.; Chinet, T.; Emile, J.F.; et al. Predictive role of plasmatic biomarkers in advanced non-small cell lung cancer treated by nivolumab. *Oncoimmunology* **2018**, *7*, e1452581, doi:10.1080/2162402X.2018.1452581.

6. Incorvaia, L.; Fanale, D.; Badalamenti, G.; Porta, C.; Olive, D.; De Luca, I.; Brando, C.; Rizzo, M.; Messina, C.; Rediti, M.; et al. Baseline plasma levels of soluble PD-1, PD-L1, and BTN3A1 predict response to nivolumab treatment in patients with metastatic renal cell carcinoma: a step toward a biomarker for therapeutic decisions. *Oncoimmunology* **2020**, *9*, 1832348, doi:10.1080/2162402X.2020.1832348.

7. Ji, S.; Chen, H.; Yang, K.; Zhang, G.; Mao, B.; Hu, Y.; Zhang, H.; Xu, J. Peripheral cytokine levels as predictive biomarkers of benefit from immune checkpoint inhibitors in cancer therapy. *Biomed Pharmacother* **2020**, *129*, 110457, doi:10.1016/j.biopha.2020.110457.

8. Krafft, U.; Olah, C.; Reis, H.; Kesch, C.; Darr, C.; Grunwald, V.; Tschirdewahn, S.; Hadaschik, B.; Horvath, O.; Kenessey, I.; et al. High Serum PD-L1 Levels Are Associated with Poor Survival in Urothelial Cancer Patients Treated with Chemotherapy and Immune Checkpoint Inhibitor Therapy. *Cancers (Basel)* **2021**, *13*, doi:10.3390/cancers13112548.

9. Mahoney, K.M.; Ross-Macdonald, P.; Yuan, L.; Song, L.; Veras, E.; Wind-Rotolo, M.; McDermott, D.F.; Stephen Hodi, F.; Choueiri, T.K.; Freeman, G.J. Soluble PD-L1 as an early marker of progressive disease on nivolumab. *J Immunother Cancer* **2022**, *10*, doi:10.1136/jitc-2021-003527.

10. Mazzaschi, G.; Minari, R.; Zecca, A.; Cavazzoni, A.; Ferri, V.; Mori, C.; Squadrilli, A.; Bordi, P.; Buti, S.; Bersanelli, M.; et al. Soluble PD-L1 and Circulating CD8+PD-1+ and NK Cells Enclose a Prognostic and Predictive Immune Effector Score in Immunotherapy Treated NSCLC patients. *Lung Cancer* **2020**, *148*, 1-11, doi:10.1016/j.lungcan.2020.07.028.

11. Tiako Meyo, M.; Jouinot, A.; Giroux-Leprieur, E.; Fabre, E.; Wislez, M.; Alifano, M.; Leroy, K.; Boudou-Rouquette, P.; Tlemsani, C.; Khoudour, N.; et al. Predictive Value of Soluble PD-1, PD-L1, VEGFA, CD40 Ligand and CD44 for Nivolumab Therapy in Advanced Non-Small Cell Lung Cancer: A Case-Control Study. *Cancers (Basel)* **2020**, *12*, doi:10.3390/cancers12020473.

12. Murakami, S.; Shibaki, R.; Matsumoto, Y.; Yoshida, T.; Goto, Y.; Kanda, S.; Horinouchi, H.; Fujiwara, Y.; Yamamoto, N.; Ohe, Y. Association between serum level soluble programmed cell death ligand 1 and prognosis in patients with non-small cell lung cancer treated with anti-PD-1 antibody. *Thorac Cancer* **2020**, *11*, 3585-3595, doi:10.1111/1759-7714.13721.

13. Okuma, Y.; Wakui, H.; Utsumi, H.; Sagawa, Y.; Hosomi, Y.; Kuwano, K.; Homma, S. Soluble Programmed Cell Death Ligand 1 as a Novel Biomarker for Nivolumab Therapy for Non-Small-cell Lung Cancer. *Clin Lung Cancer* **2018**, *19*, 410-417 e411, doi:10.1016/j.cllc.2018.04.014.

14. Oh, S.Y.; Kim, S.; Keam, B.; Kim, T.M.; Kim, D.W.; Heo, D.S. Soluble PD-L1 is a predictive and prognostic biomarker in advanced cancer patients who receive immune checkpoint blockade treatment. *Sci Rep* **2021**, *11*, 19712, doi:10.1038/s41598-021-99311-y.

15. Ugurel, S.; Schadendorf, D.; Horny, K.; Sucker, A.; Schramm, S.; Utikal, J.; Pfohler, C.; Herbst, R.; Schilling, B.; Blank, C.; et al. Elevated baseline serum PD-1 or PD-L1 predicts poor outcome of PD-1 inhibition therapy in metastatic melanoma. *Ann Oncol* **2020**, *31*, 144-152, doi:10.1016/j.annonc.2019.09.005.

16. Yang, Q.; Chen, M.; Gu, J.; Niu, K.; Zhao, X.; Zheng, L.; Xu, Z.; Yu, Y.; Li, F.; Meng, L.; et al. Novel Biomarkers of Dynamic Blood PD-L1 Expression for Immune Checkpoint Inhibitors in Advanced Non-Small-Cell Lung Cancer Patients. *Front Immunol* **2021**, *12*, 665133, doi:10.3389/fimmu.2021.665133.

17. Zhou, J.; Mahoney, K.M.; Giobbie-Hurder, A.; Zhao, F.; Lee, S.; Liao, X.; Rodig, S.; Li, J.; Wu, X.; Butterfield, L.H.; et al. Soluble PD-L1 as a Biomarker in Malignant Melanoma Treated with Checkpoint Blockade. *Cancer Immunol Res* **2017**, *5*, 480-492, doi:10.1158/2326-6066.CIR-16-0329.
